# Multiphase Structured Latent Curve Models for Count Response Data: A Re-Analysis of the Acquisition of Morphology in English

**DOI:** 10.1017/psy.2025.8

**Published:** 2025-03-18

**Authors:** Marian M. Strazzeri, Jeffrey R. Harring, Nan Bernstein Ratner

**Affiliations:** 1 Department of Human Development and Quantitative Methodology, University of Maryland, College Park, MD, USA; 2 Department of Hearing and Speech Sciences, University of Maryland, College Park, MD, USA

**Keywords:** multivariate count data, morpheme, nonlinear random effects, piecewise growth models, structured latent curve models, expressive language

## Abstract

Structured latent curve models (SLCMs) for continuous repeated measures data have been the subject of considerable recent research activity. In this article, we develop a first-order SLCM for repeated measures count data where the underlying change process is theorized to develop in distinct phases. Parameters of the multiphase or piecewise growth model, including changepoints, are allowed to vary across individuals. Exposure is allowed to vary across both individuals and time. We demonstrate our modeling approach on empirical expressive language data (grammatical morpheme counts) drawn from multiple distinct corpora available in the Child Language Data Exchange System (CHILDES), where the acquisition of grammatical morphology is understood to occur in distinct phases in typically developing children. A multiphase SLCM is fit to summarize individuals’ data as well as the average developmental pattern. Change in time-varying dispersion (unexplained variability in morpheme counts) over the course of early childhood is modeled concurrently to provide additional insights into acquisition. Unique characteristics of count data create modeling, identification, estimation, and diagnostic challenges that are exacerbated by incorporating growth models with nonlinear random effects. These are discussed at length. We provide annotated software code for each of models used in the empirical example.

## 1 Introduction

With advances in real-time data collection technology, multivariate count data are collected with increasing frequency in the measurement of a latent construct over time, where the underlying change process is often nonlinear. For example, the development of grammar (i.e., morphology and syntax) in General American English (GAE) is understood to follow a linear-linear multiphase process in typically developing children, with an initial phase of rapid acquisition occurring between 2 and 4 years of age followed by a period of more gradual, sustained development and mastery (e.g., Marchman & Bates, 1994; Zukowski & Bernstein Ratner, 2024). One popular measure of GAE morphosyntactic development involves counting the number of times various grammatical morphemes (see Table 1) are correctly used within an oral language sample. Brown (1973) posited that these 14 grammatical morphemes are acquired at different stages of GAE expressive language development that track with chronological age in typically developing children, where all 14 morphemes are usually attained by about 4 years of age.

**Table 1:** Brown’s (1973) grammatical morphemes (BGMs) in order of acquisition

Morpheme			Typical age of	Brown’s (1973)
type (item)	Morpheme	Example	acquisition (in months)	developmental stage
1	Present progressive	Baby cry**ing**.	27–30	II
2	In	Juice **in** cup.	27–30	II
3	On	Book **on** table.	27–30	II
4	Regular plural	Daddy have tool**s**.	27–30	II
5	Irregular past	Dog **ate** bone.	31–34	III
6	Possessive	Jake**’s** apple.	31–34	III
7	Uncontractible copula	This **is** mine.	31–34	III
8	Articles	**A** red apple.	35–40	IV
9	Regular past	He jump**ed** high.	35–40	IV
10	Regular third person	Susie drink**s**.	35–40	IV
11	Irregular third person	Kitty **has** a toy.	41–46	V
12	Uncontractible auxiliary	She **was** running.	41–46	V
13	Contractible copula	It**’s** cold outside.	41–46	V
14	Contractible auxiliary	Mommy**’s** crying.	41–46	V

*Note*: Adapted from Table 38 in Brown (1973) as well as Marchman & Bates (1994), Miller & Chapman (1981), and Zukowski & Bernstein Ratner (2024). For Stages II–V, Brown (1973) defined the criterion for *acquisition* of a morpheme type as when that morpheme type is used in at least 90% of “obligatory contexts” in “three successive samples” of oral language. An in-depth discussion of obligatory contexts may be found in Brown (1973), particularly pages 254–255.

Expressive language disorders may be identified when a child’s acquisition of these morphemes falls below age expectations (e.g., Calder et al., 2022; Leonard & Schroeder, 2023). However, while collecting counts of Brown’s (1973) grammatical morphemes (BGMs) has been expedited by technological advances in voice recording, transcribing, and language analysis, substantial logistical challenges remain in analyzing these data for clinical use on a large scale. Currently, a clinician must painstakingly review each language sample from each child to evaluate the number of times each BGM was used *correctly* (i.e., in an “obligatory context”; Brown, 1973).

One potentially more tractable alternative might be to use piecewise growth models (e.g., Cudeck & Harring, 2010; Kohli & Harring, 2013) to analyze change in the *frequency* and *unexplained variability* with which BGMs are used while accounting for exposure (i.e., the number of utterances defining the length of an oral language sample, which often varies across individuals and measurement occasions) and individual variability in age(s) of assessment, age of expressive language emergence, rate of grammar acquisition between 2 and 4 years of age, and the age at which a child transitions from acquisition to mastery. Speech and expressive language disorders might then be identified by evaluating whether predicted individual trajectories meaningfully deviate from the population average trajectory in typically developing children, potentially facilitating large scale diagnosis and treatment that can keep pace with data collection efforts. This quantitative approach applied to a large sample drawn from multiple corpora may also yield additional insights beyond what Brown (1973) was able to discover through his classic investigation of only three children, as current clinical expectations for the timing and ordering of children’s acquisition of BGMs continue to be based on samples typically smaller than 100 children total (e.g., Paul & Alforde, 1993; Zukowski & Bernstein Ratner, 2024).

**Figure 1:**
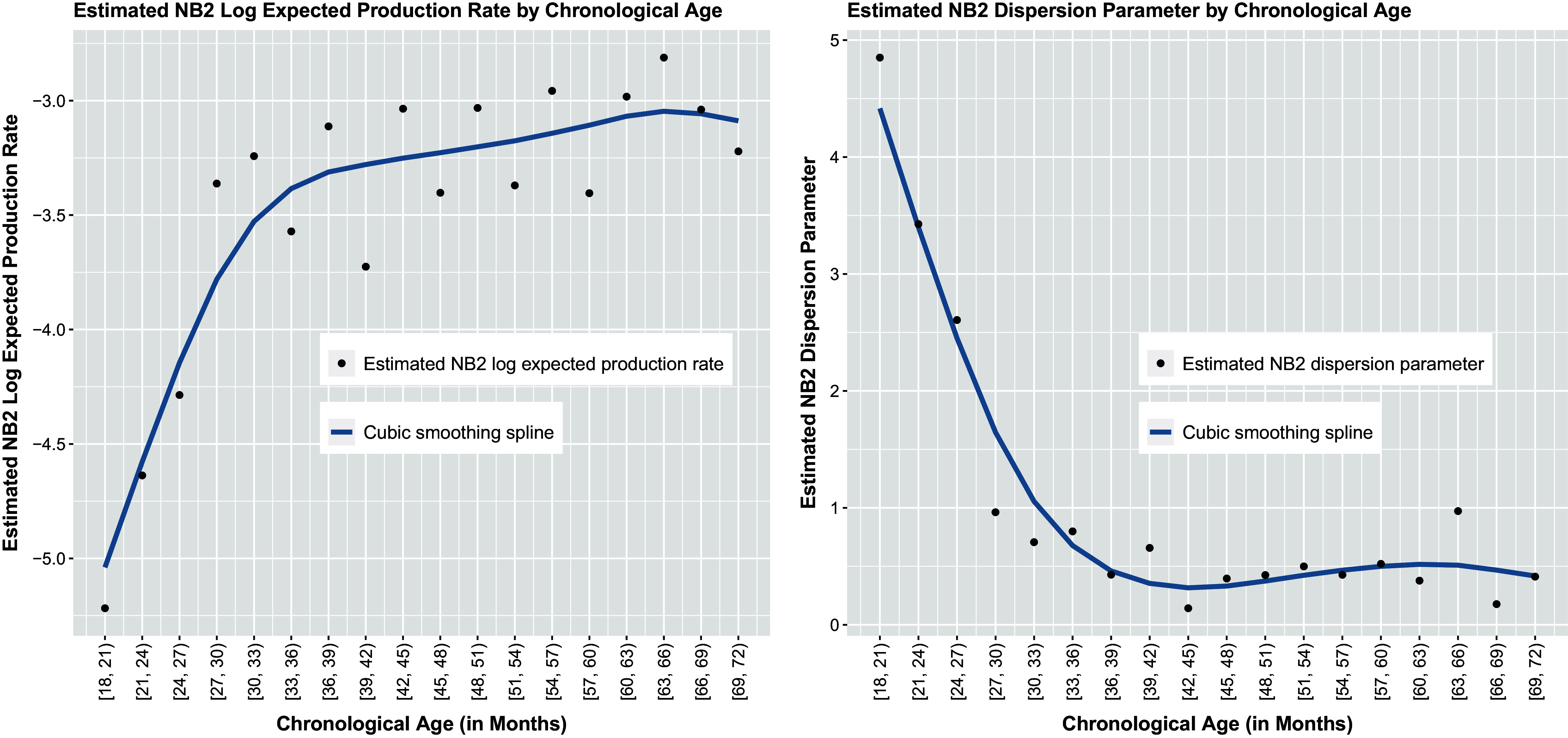
Cross-sectionally estimated NB2 log expected BGM2 production rate and dispersion parameter by chronological age. *Note*: BGM2 denotes Brown’s (1973) second grammatical morpheme, “in” (see Table 1). NB2 denotes the Negative Binomial distribution with mean 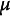 , dispersion 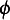 , and quadratic variance function 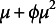 . Sample characteristics are provided in Table 3.

For example, of Brown’s (1973) 14 grammatical morphemes (Table 1), “in” is one of the first morphemes acquired by typically developing native GAE-speakers. Although production of “in” is known to be sensitive to input and language sampling context, issues with the production of “in” may foreshadow issues with both expressive language development overall and more strictly grammatical (as opposed to lexical) morphemes that are typically acquired later in childhood (e.g., Clark, 1973; Morgenstern & Sekali, 2009). Interestingly, of Brown’s (1973) 14 grammatical morphemes, production of “in” also appears to follow the most distinctly multiphasic trajectory over the course of early childhood in the combined sample of children drawn from across multiple corpora. In the left-most panel of Figure 1, one can see the *frequency* with which typically developing children produce the morpheme “in” increases rapidly between 1.5 and 3 years of age but then levels off. Simultaneously, in the right-most panel of Figure 1, *unexplained variability* in use of this morpheme drops dramatically between 1.5 and 3 years of age and then remains low. Collectively, these trajectories suggest acquisition of the morpheme “in” might be evidenced, among typically developing children, by an increase in *explained use*, which may prove to be a facile manifestation of *correct* use. Facilitating scalable clinical evaluation of the correct use of “in” may expedite early identification of broader developmental issues or predict later grammatical issues, potentially providing the opportunity for earlier intervention and better outcomes.

Processes in which change occurs in distinct phases, such as the acquisition of GAE grammar, can be modeled using piecewise or spline functions (e.g., Cudeck & Klebe, 2002; Seber & Wild, 2003). Piecewise growth models are quite flexible and can accommodate a variety of scenarios inadequately represented by mathematical functions for single-stage change processes (Grimm et al., 2011; Sterba, 2014). Fitting these models to repeated measures data that exhibit distinct phases allows one to evaluate when transitions from phase-to-phase might occur while also permitting the growth trajectory within each phase to be tailored to fit the localized data with growth parameters that directly relate to characteristics of the underlying process.

With that said, the interpretation and utility of a multiphase model and freely estimated changepoint(s) depend on the empirical context. In the evaluation of use of the morpheme “in” over the course of early childhood, quantifying the population average age of transition from an initial phase of rapid development (phase 1) to a subsequent period of more gradual, sustained development and mastery (phase 2) in typically developing children may help inform when children ought to be assessed for expressive language disorders, such as late language emergence. More specifically, if (a) typically developing children are expected to transition to slower, sustained development at a certain age and (b) the leveling off of both frequency and unexplained variability in use of the morpheme “in” at this age means acquisition of this morpheme is complete, then the population average age of transition may be a reasonable time to consider evaluating a child’s acquisition of the morpheme “in.” Assessing a child’s mastery of this morpheme too soon may result in a child being misidentified as potentially having an expressive language disorder due to the rapid development that is still occurring, while assessing too long after the population average age of transition may compromise the efficacy of targeted interventions and potential future outcomes for the child. Comparing a child’s individual trajectory and changepoint to the population average trajectory and changepoint among typically developing children may help identify children who fall below age expectations. For example, transitioning to slower, sustained development (phase 2) after the population average age may correspond to the child settling into a potentially long-term lower-than-average level of production of the morpheme “in.” For an individual morpheme, this may not mean much in terms of a child’s overall level of morphosyntactic development, but if a similar pattern is noted for other BGMs, further clinical evaluation and monitoring may be warranted.

Although several statistical frameworks exist to accommodate piecewise functions, we extend the structured latent curve model (SLCM; Browne, 1993) developed by Harring et al. (2021) to account for potentially non-monotonic, nonlinear trajectories comprised of two or more phases. These authors also demonstrated how transition times (knots, changepoints) could be freely estimated model parameters that are either held fixed or allowed to randomly vary across individuals. However, this SLCM approach has its own (mathematical rather than logistical) challenges. Longitudinal BGM counts evaluated to assess GAE morphosyntactic development, for instance, must be modeled as arising from a *counting process* to avoid challenges interpreting parameter estimates, confidence intervals, and predictions that imply negative morpheme counts, especially when counts are small, as they generally are in very young children. Computational difficulties may arise when estimating a nonlinear change process with linear and nonlinear random effects connected to observed counts via a nonlinear link function with multiple, time-varying dispersion parameters that may vary widely in magnitude and even follow their own trajectory.

The remainder of this article is divided into four major sections. First, we describe count data and how such data are generally modeled. We then introduce a first-order multiphase SLCM for count response data in which the growth parameters—including changepoints—are unknown and allowed to vary across individuals and exposure is permitted to vary across both individuals and time/assessments. Although typical acquisition of the morpheme “in” may follow a linear-linear trajectory in the empirical example, the proposed model permits non-monotonic change over the entire measurement period that may occur in more than two phases, where the functional form of change within a given phase is tailored to adequately summarize the main characteristics of the developmental process (Harring et al., 2021). We also demonstrate how to incorporate a trajectory describing concurrent change in time-varying dispersion (unexplained variability in morpheme counts) over the course of early childhood to provide additional insights into acquisition. Second, we discuss at length a number of analytic challenges and considerations surrounding model assumptions, the empirical evaluation of those assumptions, model identification, and model estimation. Third, we present the count data used in the empirical example, morpheme counts drawn from young children across multiple distinct corpora in CHILDES (MacWhinney, 2000), in greater detail. The results of an analysis of this data are presented, focusing on the interpretation of model parameters and corresponding graphical representations of typical and individual behavior. The proposed model is estimated using existing methods and software, and we highlight particular decision points as we step through the analysis. Lastly, we provide concluding remarks and discuss limitations and future directions, including how the basic SCLM can be extended to second-order growth processes.

## 2 Modeling counting processes

The development of latent growth models (LGMs) for count responses—along with corresponding estimation and fit assessment methods—has involved intertwining advancements in the statistical literature of both observed and latent variable models. We briefly review these developments and opportunities in modeling multiphasic latent growth measured by one or more count indicators assessed repeatedly over time by building the complete model from the ground up—i.e., from the observed data up to the hypothesized data-generating latent structure that is typically of primary interest, where the observed and latent variables are connected by measurement models. First, we highlight key characteristics of univariate count data and the processes by which they are generated. We then describe measurement and structural models for count response data and consider potential paths forward for extending count data LGMs to accommodate multiphasic latent trajectories.

### 2.1 Univariate count data

Count data may assume any nonnegative integer value and are typically highly skewed. Count data can be *empirically* (unconditionally) *equidispersed* when the empirical mean and variance are equal, *empirically underdispersed* when the empirical mean exceeds the empirical variance, or *empirically overdispersed* when the empirical variance exceeds the empirical mean. Realizations of a count variable may be directly observed or unobserved (latent) and may be measured with error in either case (e.g., Cameron & Trivedi, 2013). For example, a pedometer counts steps indirectly as a function of movement (so that step count is latent) and the resulting counts are subject to measurement error arising from the imperfect mapping between detected movement and steps taken. Alternatively, counts of grammatical morphemes in a video-recorded and transcribed oral language sample are directly measured with what is probably a very small amount of error. Count data are also at the ratio level of measurement defined by ordered categories with equal intervals and true zero, where the latter represents an absence of the measured count variable. This is in contrast to the more commonly analyzed ordinal data, whose numeric values, including zero, have no inherent meaning but rather indicate the relative position/ordering of the various levels of the ordinal variable. In a measurement context in which a latent variable gives rise to the observed count variable, a count of zero may represent an absence of the underlying latent variable in addition to an absence of the observed count variable, depending on what the latent variable represents and the probability distribution it is assumed to follow. Consider a latent variable representing symptom severity, for instance. In this scenario, a count of zero may represent absence of the symptom. Alternatively, for a normally distributed latent variable representing an individual’s level of expressive language development, a count of zero may correspond to levels of development falling below a certain threshold along the latent continuum.

In this article, we restrict our attention to count data arising from a single counting process, although count data from multiple response processes can be accommodated. A counting process is a stochastic process describing the nonnegative integer number of events we expect to observe within a given exposure, where the number of observed events cannot decrease with increasing exposure. The exposure quantifies the length of time, space, or number of trials over which events are recorded and must be either a positive real number or a (positive) natural number (e.g., Cameron & Trivedi, 1998, 2013; Hilbe, 2011). An exposure that may vary across observations (e.g., individuals, measurement occasions) yields an *exposure variable*. Like counts themselves, an exposure variable (here: the number of utterances sampled from a child at an assessment that defines the length of the oral language sample) may be either directly observed or latent and may be measured with error in either case. When an exposure variable cannot be measured directly, is multi-faceted, and/or is not well-defined, it can sometimes be reconstructed (possibly with error) as a function of a set of measured variables. Measurement error in counts and exposure are considered at length by Cameron and Trivedi (2013, Chapter 13).

Despite the variability and/or measurement error that are commonly present in exposure, probability distributions for count data implicitly assume an exposure that is fixed and measured consistently across observations. When a consistently measured, fixed exposure is used to collect each observation:

- All observed responses are on the same scale (a necessary condition for obtaining correct values and interpretations for model parameters, including the event rate and expected count [the mean]);
- The properties of the maximum likelihood estimators of model parameters are unaffected; and
- All variability in the observed count response variable is attributable to sampling variability arising from individual differences in model parameters (e.g., Cameron & Trivedi, 2013; Hilbe, 2011).

In count models, explicitly and properly accounting for an exposure that varies across the units/observations comprising a sample is critical to obtaining correct inferences by achieving (a) and (b) and parsing variability in (c) from sampling variability in the observed responses due to varying exposure (e.g., Cameron & Trivedi, 1998). Failing to properly account for a varying exposure will yield biased parameter estimates, standard errors, and model fit statistics—leading to incorrect inferences—as neither (a), (b), nor (c) will hold. Note, however, that including an exposure variable in a count model will only yield correct inferences if the probability of observing an event per unit exposure is constant (e.g., Cameron & Trivedi, 1998). The opportunity to explicitly incorporate an exposure variable into the probability distribution governing the counting process presents itself through the regression of the distribution mean parameter onto a set of covariates that includes the exposure.

### 2.2 Probability distributions for a single counting process

Distributions modeling a single counting process lie within the exponential family (summarized in Table 2). For each of these distributions, the mean parameter quantifies the expected response and the variance is expressed as a function of the mean parameter, possibly in addition to a dispersion or “nuisance” parameter. Depending on the distribution, the mean parameter may also represent the expected event rate (e.g., the Poisson and Negative Binomial distributions). Note that different variance functions yield different probability distributions, and different distributions (models) imply different relationships between the mean and variance. For example, the Poisson distribution (de Moivre, 1711, 1718; Poisson, 1837) implies a variance that equals the mean (*model-implied [conditional] equidispersion*). Negative Binomial (NB) distributions (e.g., Cameron & Trivedi, 2013; Hilbe, 2011) imply a variance that exceeds the mean (*model-implied [conditional] overdispersion*). Meanwhile, Katz (e.g., Katz, 1963), Double Poisson (DP; e.g., Efron, 1986), Generalized Poisson (GP; e.g., Consul & Famoye, 1992; Consul & Jain, 1973; Consul, 1989), and Conway–Maxwell–Poisson (CMP; e.g., Conway & Maxwell, 1962; Guikema & Coffelt, 2008; Huang, 2017; Minka et al., 2003; Shmueli et al., 2005) distributions can imply a variance that is less than the mean (*model-implied [conditional] underdispersion*) as well as a variance that equals or exceeds the mean. With that said, distributions other than the Poisson and Negative Binomial with quadratic variance function (denoted as NB2; see Table 2) suffer from notable limitations that have restricted their use in the literature to date.

**Table 2:** Probability distributions for a single counting process

Distribution	Exponential family	Parameters	Mean: 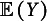	Variance: 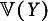
Poisson	1PEF	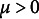	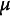	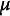
Negative Binomial (NB)	2PEF	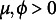	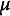	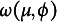
NB1	2PEF	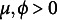	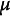	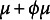
NB2	2PEF	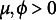	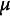	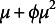
NB*p*	2PEF	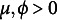	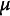	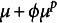
Katz family	2PEF	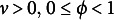	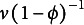	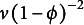
Double Poisson (DP)	2PEF	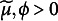	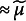	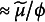
Generalized Poisson (GP)	2PEF	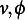	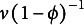	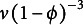
Conway–Maxwell–Poisson (CMP)	2PEF	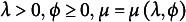		
Mean-parameterized CMP ( 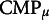 )	2PEF	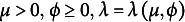	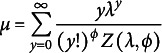	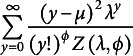

*Note*: All models are members of the exponential family and have support 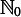 . 1PEF denotes the *one-parameter exponential family*. 2PEF denotes the *two-parameter exponential family*. *Y* denotes the count response variable. 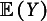 denotes the model-implied (conditional) mean. 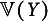 denotes the model-implied (conditional) variance, which is also called the *variance function* as the variance is a function of the mean. For all models, 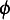 denotes the dispersion or “nuisance” parameter, where the interpretation and parameter space of 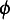 depends on the distribution. In the Poisson, NB, and 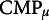 distributions, 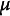 is the mean parameter. In the DP distribution, the parameter 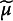 is “similar to the mean parameter” (Cameron & Trivedi, 2013). In the CMP and 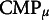 distributions, 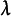 is the rate parameter. For the NB family of distributions, the variance is a function of the mean and dispersion parameters (i.e., 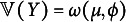 ). NB1 denotes the Negative Binomial distribution with linear variance function 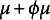 , NB2 denotes the Negative Binomial distribution with quadratic variance function 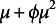 , and NB*p* denotes the Negative Binomial distribution with variance function 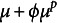 .

### 2.3 Regression models

Incorporating a regression model for the mean parameter of a counting process distribution permits the expected response to be a function of exposure and other predictors. For counting processes in the two-parameter exponential family (2PEF), a regression model for the dispersion parameter may also be specified, where the total set of regression equations specified may or may not have predictors or coefficients in common. Regression models for a count response arising from a single counting process distribution for which an exact expression for the mean response is available, such as the Poisson and NB distributions (see Table 2), can be formulated within a generalized linear model (GLM), generalized nonlinear model (GNLM), generalized linear mixed model (GLMM), or generalized nonlinear mixed model (GNLMM) framework, depending on whether the relations among predictors are linear or nonlinear and whether the coefficients of the predictors are fixed, random, or some combination thereof (e.g., Agresti, 2013; Cameron & Trivedi, 2013; Fitzmaurice et al., 2011; Hilbe, 2011; McCullagh & Nelder, 1989; Nelder & Wedderburn, 1972; Vonesh, 2012). With that said, few examples exist in the literature of a nonlinear growth function of linear and nonlinear random effects connected to observed counts via a nonlinear link function due to the computational difficulties that may arise in model estimation.

For a GLM, GNLM, GLMM, or GNLMM regressed on the mean parameter of a counting process distribution: (1) the systematic component may include an *offset*—the natural log of an exposure variable with corresponding regression coefficient fixed at one; (2) the random component is parameterized in terms of the mean and dispersion of the counting process (see Table 2); and (3) a natural log link function is used to connect the random and systematic components. Although other link functions that ensure the mean is always positive may be used with count responses (e.g., Wedel et al., 2003), the natural log link function is generally preferred as it is the canonical link function for the Poisson mean parameter. Note that including an offset in the systematic component of the regression on the mean response allows the mean to be expressed as the product of exposure and the hazard rate quantifying the expected count per unit exposure, where either or both may be functions of observed and/or latent variables (e.g., Cameron & Trivedi, 2013).

Note that one may observe equidispersion, overdispersion, or underdispersion under a fitted model when the empirical (observed) variance equals, exceeds, or is less than the model-implied (conditional) variance, respectively. (The discerning reader may observe the distinction between under-, equi-, and overdispersion that is empirical, model-implied, or arising when a model is fit to data is rarely made explicit in the literature.) The presence of under- or overdispersion is understood to yield incorrect standard errors, incorrect model fit statistics, and, as a result, fallacious inferences with real, practical implications (e.g., Cameron & Trivedi, 2013; Hilbe, 2011). Under- or overdispersion arising from model misspecification may additionally yield incorrect inferences about the response process but can be addressed through careful reconsideration of the systematic component of the regression model for the mean response and subsequent selection of a more appropriate conditional probability distribution (see Table 2).

Given the computational challenges involved in implementing other counting process distributions enumerated in Table 2, Poisson and NB2 distributions remain popular choices across a variety of scientific applications. Furthermore, given the frequency with which overdispersion is observed in practice, NB2 models present an attractive choice for use in modeling change processes measured by the repeated measurement of a single count variable over time. Using a conditional response distribution in the 2PEF additionally permits investigation of the joint behavior of the mean response and unexplained variability among responses (manifesting as overdispersion) over time by fitting a first-order LGM to the mean responses and a separate trajectory (that need not be a GLM or GNLM) to the time-varying dispersion parameters. As such, although the proposed first-order LGM may be specified using any counting process distribution in the exponential family, we illustrate our approach using the NB2 distribution.

### 2.4 First-order multiphase SLCM for count data

Typically, first-order LGMs are those that model a single observed indicator repeatedly measured at a set of time points or occasions for a sample of individuals. We begin this section by explicating the notation used to define the response data, the measurement occasions, and individuals. For additional clarity, we couch explication of the notation in the empirical example of modeling counts of Brown’s (1973) second grammatical morpheme (BGM2) “in” (see Table 1) collected in the longitudinal assessment of GAE morphosyntactic development over the course of early childhood (Figure 1).

**Figure 2:**
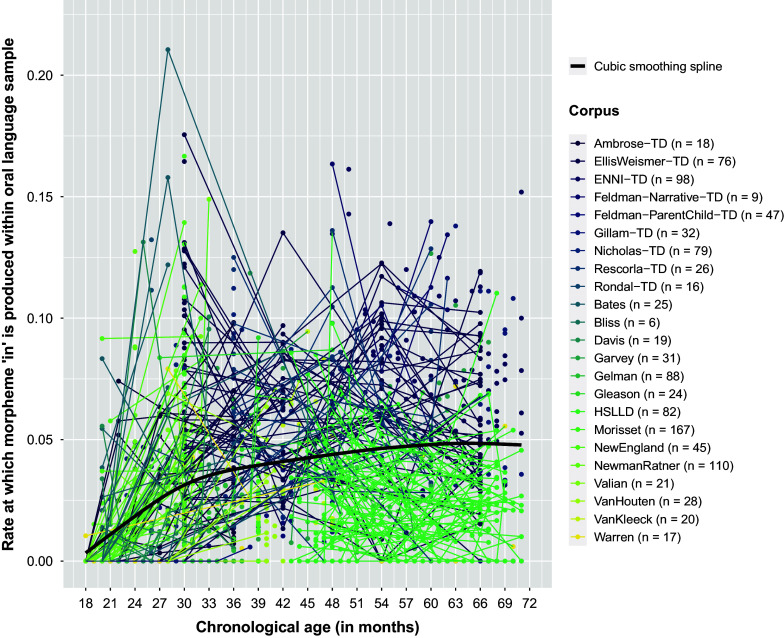
Rate at which the morpheme “in” is produced within an oral language sample by chronological age and corpus. *Note*: Brown’s (1973) grammatical morphemes are summarized in Table 1. Sample characteristics are provided in Table 3.

**Figure 3:**
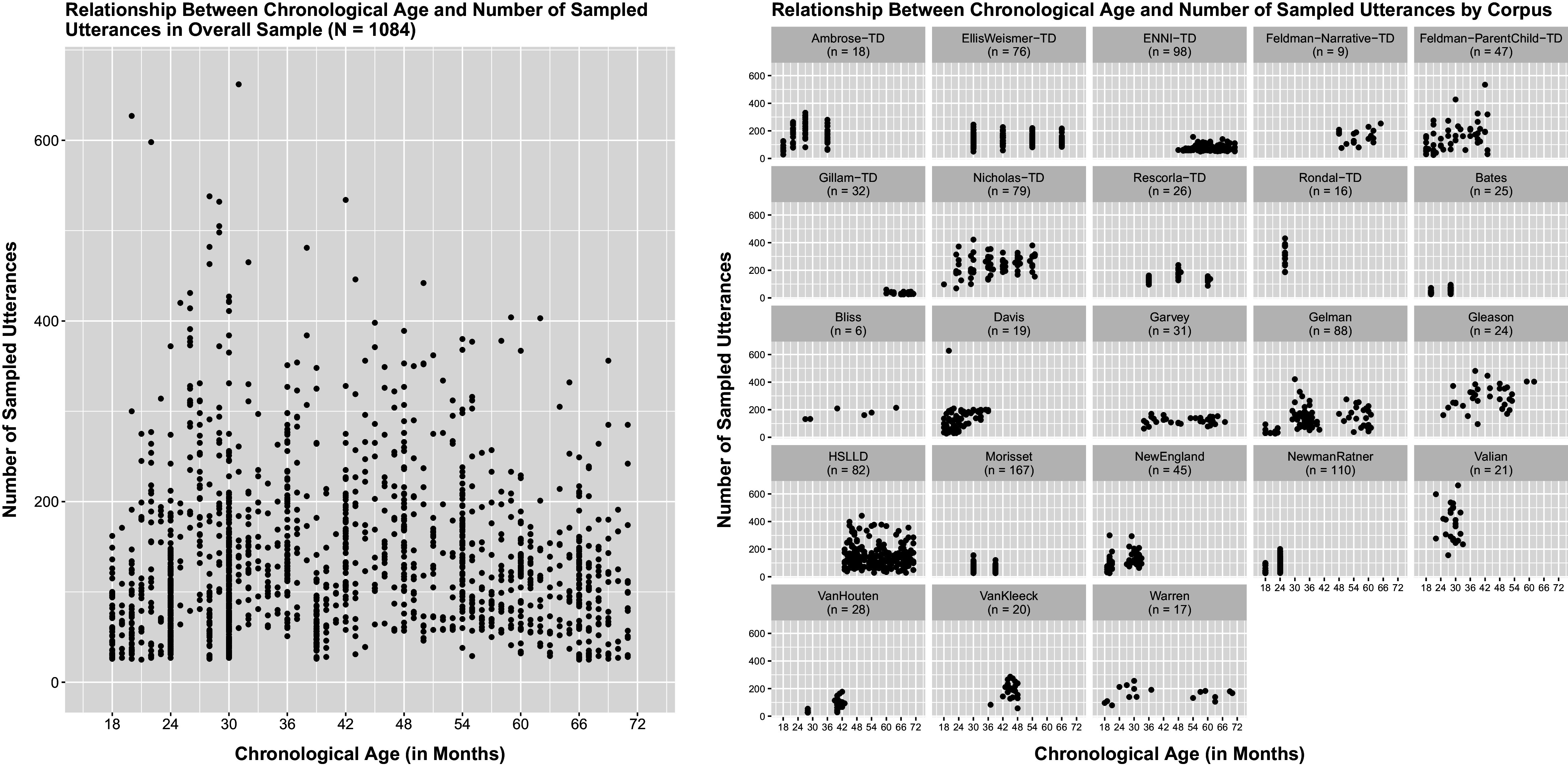
Relationship between chronological age and number of sampled utterances. *Note*: Sample characteristics are provided in Table 3.

Let 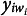 denote the number of “in” morphemes produced by child *i* out of 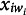 utterances sampled at chronological age 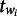 months. As implied by the notation, the chronological ages (in months) at which children were assessed varied across children both within and among corpora (see Table 3 and Figure 2). This is because, in each corpus, oral language was sampled at certain, planned chronological ages that differed across corpora, and children *within* a corpus were sometimes also assessed at slightly different ages than planned and/or were missing planned assessments.^1^ As implied by the notation 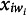 , the number of sampled utterances (exposure) varied also across children and assessments (Figure 3). A spaghetti plot of the rates at which the morpheme “in” is produced within an oral language sample in the overall sample of children is given in Figure 2 with a lowess smooth of the mean function superimposed.

**Table 3:** Sample characteristics

					Child’s reported sex (%)				
CHILDES Collection	Corpus	Activity	DOI	*n*	Male	Female	NR	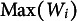	Chronological ages (months)
Clinical-Eng	Ambrose-TD	TP	10.21415/T56P63	18	44.44	55.56	0.00	4	[18, 36]
Clinical-Eng	EllisWeismer-TD	TP	10.21415/T5FP4H	76	51.32	46.05	2.63	1	[30, 66]
Clinical-Eng	ENNI-TD	N	10.21415/T51G7V	98	48.98	51.02	0.00	4	[48, 71]
Clinical-Eng	Feldman-Narrative-TD	N	10.21415/T5N894	9	0.00	0.00	100.00	3	[48, 65]
Clinical-Eng	Feldman-ParentChild-TD	TP	10.21415/T5GK55	47	31.91	61.70	6.38	3	[18, 43]
Clinical-Eng	Gillam-TD	N	10.21415/T5QS3N	32	40.62	59.38	0.00	1	[60, 71]
Clinical-Eng	Nicholas-TD	TP	10.21415/T50604	79	50.63	49.37	0.00	1	[18, 55]
Clinical-Eng	Rescorla-TD	TP	10.21415/T59D5W	26	92.31	3.85	3.85	3	[36, 61]
Clinical-Eng	Rondal-TD	TP	10.21415/T5RC93	16	75.00	25.00	0.00	1	[26, 26]
Eng-NA	Bates	TP	10.21415/T56W31	25	52.00	48.00	0.00	2	[20, 28]
Eng-NA	Bliss	TP	10.21415/T58W28	6	33.33	66.67	0.00	1	[27, 64]
Eng-NA	Davis	TP	10.21415/T5CW26	19	47.37	52.63	0.00	7	[18, 36]
Eng-NA	Garvey	G	10.21415/T56W2N	31	54.84	45.16	0.00	1	[34, 67]
Eng-NA	Gelman	N	10.21415/T5X69X	88	48.86	51.14	0.00	1	[18, 61]
Eng-NA	Gleason	TP, M	10.21415/T5101R	24	58.33	41.67	0.00	3	[25, 62]
Eng-NA	HSLLD	TP, M, N, B, NR	10.21415/T5H88H	82	48.78	51.22	0.00	5	[43, 71]
Eng-NA	Morisset	TP	10.21415/T53895	167	29.94	27.54	42.51	1	[30, 39]
Eng-NA	NewEngland	TP	10.21415/T52P6V	45	48.89	51.11	0.00	2	[19, 33]
Eng-NA	NewmanRatner	TP	10.21415/T5QW3P	110	47.27	52.73	0.00	2	[18, 24]
Eng-NA	Valian	TP	10.21415/T5ZS3T	21	38.10	61.90	0.00	2	[22, 33]
Eng-NA	VanHouten	TP, M	10.21415/T5Z014	28	60.71	39.29	0.00	2	[28, 43]
Eng-NA	VanKleeck	TP	10.21415/T54883	20	65.00	35.00	0.00	1	[37, 48]
Eng-NA	Warren	TP	10.21415/T51G6G	17	52.94	47.06	0.00	1	[18, 70]
			***Overall Sample:***	1,084	46.86	45.20	7.93	7	[18, 71]

*Note*: CHILDES denotes the Child Language Data Exchange System (MacWhinney, 2000); DOI denotes Digital Object Identifier; *n* denotes the number of children sampled from the indicated corpus; NR denotes Not Reported; and 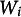 denotes the number of measurements taken over time from individual (child) *i*. In the Activity column, TP denotes oral language data were collected through engagement in a *toy play* activity, G denotes a *group* activity, M denotes a *meal* activity, B denotes a *book* activity, and N denotes a *narrative* activity. Samples drawn from listed corpora contained typically developing (TD) monolinguistic native speakers of General American English (GAE) aged [18, 72) months (i.e., aged [1.5, 6) years) with at least 25 Mean Length of Utterance (MLU)-eligible sampled utterances.

Let 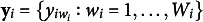 , 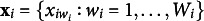 , and 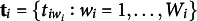 denote the sets of observed responses (BGM2 counts), exposures (numbers of sampled utterances), and measurement times (chronological ages of assessment in months), respectively, collected from the repeated measurement of a single count item/indicator (i.e., the number of “in” morphemes produced) over the course of 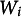 occasions for individual *i*, where the observed counts are nonnegative integers and the exposures and measurement times are positive real numbers. The number of assessments per child ( 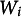 ) ranged from one to seven, inclusive, with corresponding ages of assessment ( 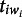 ) ranging from 18 to 71 months, inclusive (Table 3). Chronological age of assessment was binned into 3-month assessment windows (intervals), yielding a total of 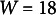 unique measurement occasions in the overall sample of 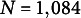 children: [18,21), [21,24), [24,27), [27,30), [30,33), [33,36), [36,39), [39,42), [42,45), [45,48), [48,51), [51,54), [54,57), [57,60), [60,63), [63,66), [66,69), and [69,72). In the analysis dataset, these measurement windows were coded using the lower bound of each 3-month interval, centered at age 18 months, yielding 

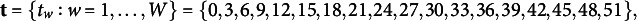

so that *w* indexes unique measurement occasion (unique chronological age of assessment) within the overall sample of *N* individuals. As such, data collected within a given interval are treated as though collected at the chronological age indicated by the lower bound of that interval in the analysis and interpretation of model parameters. For example, BGM2 counts collected at ages [18, 21) months are treated as though collected at age 18 months. Since not all children were assessed within each 3-month interval, the ages of assessment for child *i* ( 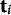 ) are a subset of the unique ages of assessment in the overall sample ( 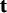 ), such that 

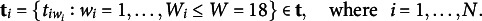



If a child had more than one assessment within a 3-month interval, one of those assessments was randomly selected for analysis so that each child contributed at most one assessment per interval (i.e., data are cross-sectional within each 3-month assessment window). Note that the use of age intervals spanning a few months is a fairly common practice in the clinical assessment of expressive language development because of dynamic and rapid growth in grammar acquisition during the earliest stages of child language development (e.g., Carrow-Woolfolk, 2011; Leadholm & Miller, 1992; Miller & Chapman, 1981; Pavelko & Owens, 2017; Rice et al., 2010; Scarborough et al., 1991; Sparrow et al., 2016). Here, *3-month* intervals were used in order to balance (a) choosing intervals small enough to maximize the amount of longitudinal data taken from each child, the number of unique measurement occasions in the overall sample, and granularity with respect to chronological age with (b) choosing intervals large enough to contain enough children to permit the estimation of dispersion *across* children within each age interval.

#### 2.4.1 Measurement model

**Figure 4:**
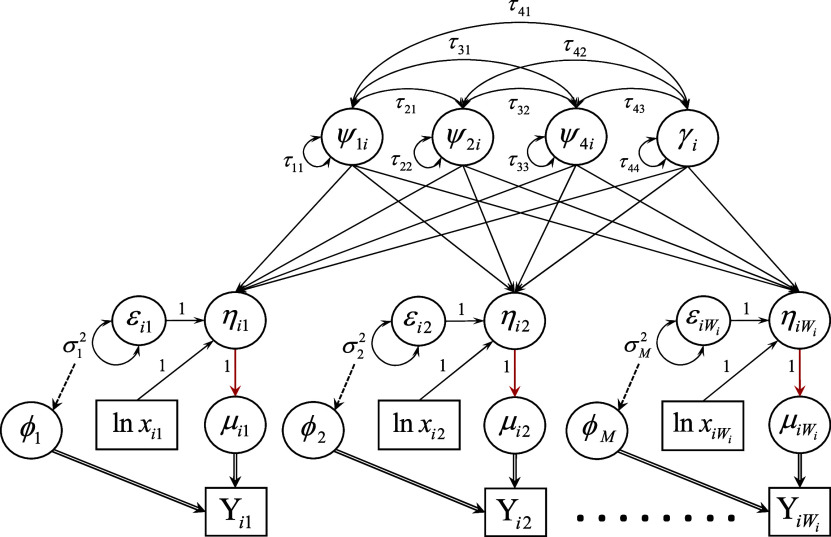
Path diagram for a first-order linear–linear latent growth model fit to repeated measurements of a single count response variable that conditionally follows a distribution in the two-parameter exponential family at each measurement occasion. *Note*: A solid, single-line, black arrow indicates a structural relationship with an identity link function. A solid, single-line, red arrow indicates a structural relationship with a non-identity (e.g., natural log) link function. A dashed black arrow from *A* to *B* indicates *A* gives rise to *B* directly and/or indirectly. A solid, double-line, black arrow from *A* to *B* indicates *A* generates *B*.

Due to the myriad of components unique to the modeling of count response data as well as the complexity of the notation, we present an example of the proposed first-order multiphase LGM as a path diagram in Figure 4. Following the typical structural equation modeling convention for path diagrams, squares represent observed variable indicators, circles represent latent variables, single-headed arrows denote directed relations among observed and latent variables, and double-headed arrows denote variances of and covariances between variables. Other notation germane to the explication of the measurement and structural components of the LGM in Figure 4 is detailed and explained shortly.

Suppose 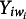 measures individual *i*’s level of target construct 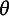 (e.g., level of morphosyntactic development) at time 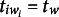 , where corresponding observed response 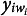 is generated from a counting process distribution in the 2PEF with density 
(1)

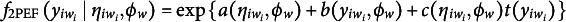

with time-specific dispersion parameter 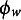 and natural/canonical parameter 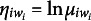 , where 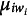 represents individual *i*’s expected response at occasion 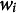 . For the empirical example, we use the 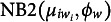 density in Equation (2). 
(2)

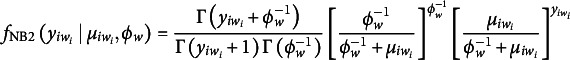



Individual *i*’s expected response at chronological age 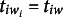 months is expressed as a function (Equation (3)) of exposure 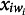 , individual *i*’s levels of the target construct 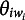 , item intercept 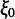 , item slope 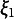 , and error 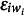 . 
(3)

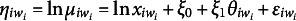

Note the subscript 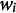 in 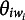 indicates Equation (3) applies to those chronological ages at which child *i* is actually assessed, though the child’s latent construct exists continuously across all chronological ages (including 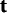 ), measured or not, so that 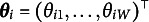 . The item intercept quantifies the *log* expected response (expected count) per unit exposure at the population average level of the target construct (i.e., when 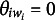 ), while the item slope captures the strength of the (positive) linear relation between 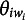 and log expected response, 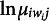 . Note that the expected count per unit exposure increases multiplicatively by an order of 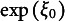 as 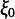 increases, holding 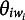 constant. Also note that item parameters 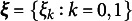 do not vary across individuals or measurement occasions to achieve measurement invariance.

The error, 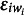 , in the linear predictor of the mean response in Equation (3) may be non-zero due to the omission of important predictors of the mean response, misspecification of the structural model, and/or measurement error in the offset, 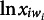 . This error propagates down to the observed data level manifesting as overdispersion (unexplained variability) in the observed responses (e.g., Cameron & Trivedi, 2013). The set of time-specific errors for individual *i*, 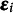 , is normally distributed with zero mean vector and covariance matrix 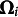 . 

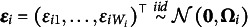

The set of time-specific latent constructs for individual *i* follows a different multivariate normal distribution with mean vector 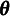 and covariance matrix 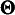 . 

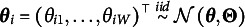



#### 2.4.2 Structural model

Change in an individual’s level of the target construct over time follows a theoretically defensible growth model, *f*, expressed as a function of measurement times, 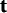 , and individual latent growth factors, 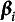 . 
(4)

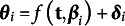



The set of disturbances, 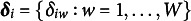 , in Equation (4) represents the set of time-specific regression errors induced by misspecifying the true (data-generating) trajectory of 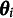 over 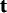 . This could occur by omitting predictors of an individual’s level of the target construct and/or misspecifying the functional form of *f*. The disturbances are assumed to jointly follow a multivariate normal distribution with zero mean vector and covariance matrix 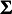 . 

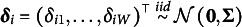



In Equation (5), we assume that *f* is a piecewise growth model having the general form, 
(5)

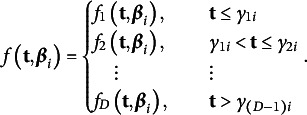

The trajectory in each of the 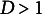 phases may have a distinct functional form that need not be a polynomial. Moreover, the trajectory need not be monotonic within a phase nor over the entire measurement period. The changepoints (a.k.a., *join points* or *knots*, denoted by 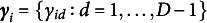 ) indicate the times of transition from phase to phase. These transition times may be unknown (i.e., parameters to be estimated from the data) and may vary across individuals (i.e., have random effects). The transition from one phase to the next may be discontinuous (e.g., a jump up or a drop down), continuous but abrupt (zero-order continuity), or continuous and gradual/smooth (first-order continuity or greater, where higher orders of continuity correspond to greater degrees of smoothness).

Individual growth factors 
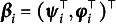
 include *p* parameters, 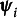 , that enter the function in Equation (5) linearly and *q* parameters, 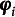 , that enter the function in a nonlinear manner. Here, we define a growth parameter as being *linear* if the first partial derivative of *f* taken with respect to the growth parameter does *not* include the parameter. Alternatively, a growth parameter is considered to be *nonlinear* if the first partial derivative of *f* taken with respect to the growth parameter *includes* the parameter. Nonlinear growth factors include—but need not be limited to—unknown, individual-specific changepoints.

Individual linear and nonlinear growth factors may each be expressed as a linear combination of population growth parameters governing the population average trajectory—linear and nonlinear fixed effects 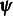 and 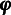 —and individual *i*’s linear and nonlinear random effects, 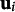 and 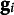 , respectively. Random effects 
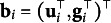
 are assumed to jointly follow a multivariate normal distribution with zero mean vector and symmetric covariance matrix **T**. 
(6)

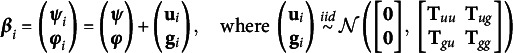

Note that elements of **T** may be constrained at zero for theoretical but also potentially computational (pragmatic) reasons.

Following S. A. Blozis & Harring (2016) and S. A. Blozis & Harring (2017), the growth function for individual *i* in Equation (5) can be reformulated as a SLCM defined by a first-order Taylor series expansion taken with respect to the parameters of the mean growth function and linearly weighted by a set of individual-specific weights (i.e., random effects, 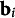 ). 
(7)

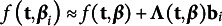



The columns (i.e., *basis functions*) of 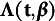 are the the first-order partial derivatives of the mean (i.e., target) function, 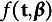 . 
(8)

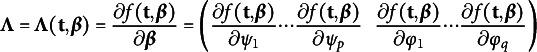



As a SLCM, 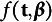 is assumed to be invariant to a constant scaling factor (see Shapiro & Browne, 1987, Condition 2). Consequentially, there is a set of parameters, denoted here by 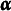 , such that 
(9)

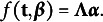



Because 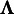 is the set of first-order partial derivatives of 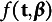 taken with respect to 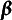 (see Equation (8)), elements of parameter vector 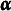 can be obtained by solving the linear equations in Equation (9). It turns out that solving these linear equations results in setting all parameters in 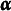 that enter nonlinearly to 0 (i.e., 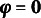 ). This permits the recovery of the target function (Preacher & Hancock, 2015). Then in the individual-level model in Equation (7), 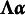 can be substituted for the mean function, 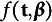 . Thus, the individual-level model can be re-expressed as 

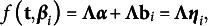

where 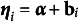 .

Note that when a single count indicator is measured at each occasion, the item intercept is constrained at zero 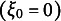 and item slope at one 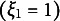 to achieve model identification while preserving meaningful interpretation of the growth parameters. 
(10)

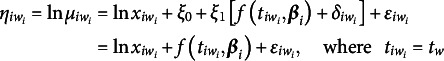



Additionally, regression disturbance 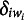 is absorbed into measurement error 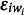 , impacting the interpretation of 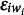 and any resulting overdispersion. 

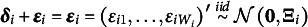



#### 2.4.3 Dispersion trajectory

If a conditional distribution in the 2PEF, such as the NB2 distribution, is utilized for the count response measured repeatedly over time, the magnitude of conditional (model-implied) dispersion may notably vary over time and possibly even follow it’s own trajectory. Let 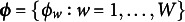 denote the complete set of freely estimated dispersion parameters corresponding to the *W* unique measurement times 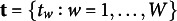 in the overall sample of *N* individuals. Where appropriate, one may fit a trajectory with coefficients 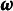 to time-varying dispersion parameters 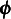 to describe change in dispersion over time. 
(11)

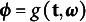



The trajectory fit to the time-specific dispersion parameters need not be a GLM nor utilize polynomial growth, but whatever function is ultimately used, it must capture the essential characteristics of the freely estimated dispersion parameters across time. For example, in modeling counts of Brown’s (1973) second grammatical morpheme (BGM2) “in” (see Table 1) collected in the longitudinal assessment of GAE morphosyntactic development over the course of early childhood (Figure 1), change in model-implied overdispersion over time might be described by a linear function fit to the natural log of the NB2 dispersion parameters 
(12)

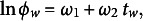

where 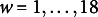 and 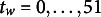 . Alternatively, a more precipitous decline over the first several months of early childhood might be achieved through an exponential decay function with a nonzero asymptote (see Equation (13)) to describe change in dispersion over time, where 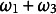 quantifies the dispersion parameter at age 18 months when 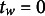 , 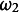 is the decay factor such that the NB2 dispersion parameter decreases by 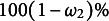 with every 1 month increase in chronological age after age 18 months, and 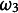 is the nonzero asymptote quantifying the NB2 dispersion parameter as children age beyond early childhood, 
(13)

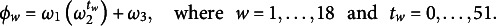



Note that the dispersion parameters are time-specific (as indicated by the subscript *w*) but do *not* vary across individuals (as indicated by the omission of *i* from the subscript). As such, any trajectory fit to the dispersion parameters exists only at the population level and is specified by imposing constraints on the time-varying dispersion parameters, reducing the number of freely estimated dispersion-specific parameters from *W* to the length of 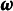 . As such, fitting a trajectory to the dispersion parameters may convey notable parsimony in addition to the ability to describe a change process of substantive interest, where gains in parsimony for a given function *g* increase as the number of unique measurement occasions (*W*) in the overall sample of *N* individuals increases.

## 3 Analytic considerations

When fitting a statistical model to a set of data, one must ensure the model is identifiable and that assumptions about the data generating process implied by the model are both theoretically defensible and reasonably satisfied based on empirical evidence generated through model fit assessment. First, we enumerate the assumptions implied by the proposed first-order multiphase SLCM. Second, we summarize salient approaches to evaluating the fit of latent variable models for count responses. Third and lastly, we discuss necessary conditions to ensure the model is *overidentified* (has more observations than free parameters) to obtain a unique set of parameter estimates and permit meaningful evaluation of model fit.

### 3.1 Model assumptions

As with any fully parametric LGM in which parametric probability distributions are assumed for both the latent variables (random effects) and observed variables (indicators/items), the following assumptions are implied when fitting the proposed first-order multiphase SLCM to longitudinal count data. First, it is assumed that the model is correctly specified (e.g., Agresti, 2013; Cameron & Trivedi, 1998, 2013; Hilbe, 2011; McCullagh & Nelder, 1989; McNeish & Kelley, 2019; Vonesh, 2012; Woods & Thissen, 2006), including correct specification of:

1. The joint distribution of the random effects;
2. The fixed and random effects included in the linear predictor of the mean response and the relations among them;
3. The conditional distribution assumed for the response; and
4. The link function connecting the mean response to its linear predictor.

Additionally, several assumptions are made about the target population and sample of individuals from whom the count responses are collected. First, the sample from which model parameters are to be estimated is assumed to be both *homogeneous* (i.e., all sampled individuals come from the same population; OECD, 2004) and *representative* (i.e., the sample is selected probabilistically and the composition of the sample is “typical” of the population with respect to certain, specified characteristics of interest upon which inferences will be based; OECD, 2002). Second, sampled individuals are assumed to be independent and sampled measurements (count responses) are assumed to be conditionally independent within an individual (e.g., McCulloch, 2003; Vonesh, 2012; Woods & Thissen, 2006).

Several measurement-specific assumptions are also made. First, measurement invariance is assumed across individuals and occasions. Second, the IRT/IFA assumption of *monotonicity* applies here, which posits that the probability of endorsing a given response category or higher increases as the level of the latent construct measured by the item increases. For a count response, the assumption of monotonicity implies that the expected response (expected count) increases as the level of the latent construct increases. Third, as measurement error is not the focus of this research, it is assumed that count responses and exposures are directly measured with, at most, minimal error that is uncorrelated with predictors of the mean response (e.g., Cameron & Trivedi, 2013).

Lastly, combining the model-, sample-, and measurement-specific assumptions enumerated above, we assume errors are uncorrelated (mutually independent) among individuals at a given measurement time as well as across occasions within each individual after conditioning on the growth trajectory, so that 
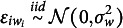
 in Equation (10) at time 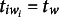 (such as is shown in Figure 4) and 

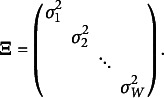



### 3.2 Model fit assessment

The goal of model fit assessment is to identify model assumptions that appear to be notably violated based on available empirical data (that are, hopefully, homogeneous and representative of the target population). Identifying sources of model-data misfit can, in conjunction with theoretical considerations, inform re-specification of the model such that more valid—and therefore more useful—inferences about the data-generation process may be drawn.

#### 3.2.1 Global fit

For latent variable models fit to count response data, likelihood-based measures of relative overall model-data fit—such as the Likelihood Ratio Test (LRT) for comparing nested models and the Akaike Information Criterion (AIC; Akaike, 1974) and Bayesian Information Criterion (BIC; Schwarz, 1978) for comparing non-nested models—have been the most commonly utilized tools, to date, for detecting various sources of misspecification, such as misspecification of the structural model (e.g., Magnus & Thissen, 2017; Man & Harring, 2019; Wedel et al., 1993), misspecification of the measurement model (e.g., Forthmann et al., 2020; Hung, 2012; Magnus & Thissen, 2017), and violations of measurement invariance (e.g., Baghaei & Doebler, 2019; Jansen, 1995). Likelihood-based measures of relative overall model-data fit have also been used in the GLM/GLMM literature to detect misspecification of: (a) the number of random effects and their joint distribution (e.g., Dean & Nielsen, 2007; Vonesh, 2012); and (b) the conditional response distribution, link function, and systematic component (e.g., Cameron & Trivedi, 1998, 2013; Hilbe, 2011; Vonesh, 2012).

#### 3.2.2 Item fit

For latent variable models fit to count response data, targeted evaluation of whether the measurement model is correctly specified has largely centered around graphical and numerical comparisons of the empirical and model-implied marginal item response distributions (e.g., Baghaei & Doebler, 2019; Forthmann et al., 2020; Magnus & Thissen, 2017; Verhelst & Kamphuis, 2009). Visual inspection of overlaid plots (e.g., histograms, density plots) and/or side-by-side numeric summaries of the empirical and model-implied marginal response frequencies for an item (e.g., Forthmann et al., 2020; Magnus & Thissen, 2017; Verhelst & Kamphuis, 2009) can yield information not just about whether the assumed measurement model appears to be correct but also how it may be wrong (e.g., help detect under- or overdispersion and excess zeros). As such, these analyses can provide insights into absolute item fit, albeit at the marginal item response level. Numeric measures of alignment between two distributions described in the statistical literature—such as the Kullback–Leibler divergence (KLD; Kullback & Leibler, 1951) and Jensen–Shannon divergence (JSD) or distance—though not used with latent variable models for count responses to date, might provide measures of relative fit at the marginal item response level by quantifying recovery of each empirical marginal item response distribution for use in model comparison.

However, evaluating the extent to which a fitted model recovers each empirical marginal item response distribution does not provide clarity regarding the source(s) of misspecification, such as the random and fixed effects included in the linear predictor, the structural model to which the latent variables are tied, the conditional item response distribution, or the link function. Fortunately, more informative targeted diagnostics have been developed within the GLM/GLMM literature for detecting misspecification of the link function (e.g., Cheng & Wu, 1994); conditional under-, equi-, or overdispersion (e.g., Breslow, 1990; Cameron & Trivedi, 1998, 2013; Hilbe, 2011; Lambert & Roeder, 1995), such as the Pearson statistic; misspecification of the variance function assumed for the NB distribution (Hilbe, 2011); and misspecification of the conditional moments (e.g., Cameron & Trivedi, 1998). Meanwhile, the evaluation of monotonicity has centered around visual inspection of estimated item slopes and corresponding standard errors as well as graphical representations of item characteristic curves (ICCs). Lastly, although not utilized in our empirical example nor in the broader literature on LVMs for count responses to date, various numerical and graphical methods in the GLMM literature might be adapted to identify individuals whose response patterns suggest the calibration sample is not homogeneous and representative (i.e., to evaluate *person fit*).

### 3.3 Model identification

The mean structure of the proposed first-order multiphase SLCM fit to count responses is comprised of all freely estimated population growth parameters in 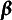 . For conditional response (counting process) distributions in the one-parameter exponential family (e.g., the Poisson distribution), the covariance structure is comprised of all freely estimated, unique growth factor variances and covariances (freely estimated unique elements of 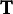 ). For conditional response distributions in the 2PEF (e.g., the NB2 distribution), the covariance structure additionally includes occasion-specific dispersion parameters 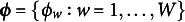 or, if a growth trajectory is imposed on 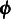 , the parameters of said trajectory (i.e., 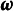 ). Note that the elements of factor loading matrix 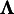 are not freely estimated but rather are functions of measurement times 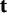 and freely estimated population growth parameters in 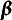 . Likewise, expected counts 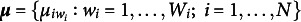 , linear predictors of the mean response 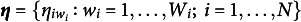 , and error variances 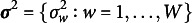 are part of the hypothesized model but are not freely estimated model parameters.

Since the proposed first-order SLCM for count responses is a CFA model with a mean structure (e.g., Browne, 1993; Kline, 2016), the number of observations is 
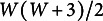
 , where, as previously noted, *W* is the number of observed (count) variables or, equivalently for this model, the number of unique measurement occasions in the overall sample of *N* individuals (e.g., Kline (2016, Rule 15.5)). Additionally, since “the identification status of a mean structure must be considered separately from that of the covariance structure” (Kline, 2016), the mean and covariance structures must each be overidentified in order for the model as a whole to be overidentified. To ensure the mean structure of the proposed model is overidentified, the total number of unique measurement occasions *W* in the sample of *N* individuals must exceed the number of freely estimated population growth parameters in 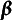 (e.g., Kline, 2016). Likewise, to ensure the covariance structure is overidentified, 
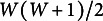
 must exceed the number of freely estimated unique elements of 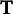 and—for conditional response distributions in the 2PEF—the number of occasion-specific dispersion parameters (*W*) or dispersion-specific regression coefficients.

### 3.4 Model estimation

The estimation of latent variable models (and GLMMs) for count responses can be challenging because each variable in the systematic component for the mean response has a nonlinear relationship with the conditional mean due to the use of a non-identity link function (e.g., Olsen & Schafer, 2001; Vonesh, 2012). As a result, there is generally no closed form solution to either the marginal log-likelihood or marginal moments, so that contemporary model estimation approaches aim to either:

1. Approximate the marginal moments of an approximate quasi-likelihood function or approximate the marginal quasi-likelihood function corresponding to specified first- and possibly also second-order conditional moments through linearization (via Taylor series expansion);
2. Maximize the marginal log-likelihood via numerical integration or simulation; or
3. Approximate the posterior distribution for the model parameters given the observed data via fully Bayesian approaches, where—like the log-likelihood—the posterior distribution typically does not have a closed form solution.

These different estimation strategies have different analytic objectives and require different assumptions. A thorough treatment of these various approaches to model estimation may be found in, for example, Vonesh (2012), Bolker et al. (2009), and Hoff (2009).

Of these different approaches, the most popular by far for latent variable models for count responses has been marginal maximum likelihood (MML) estimation implemented via numerical integration (e.g., Beisemann, 2022; Beisemann et al., 2024; Forthmann & Doebler, 2021; Forthmann et al., 2020; Hung, 2012; Jansen, 1994, 1995; Jansen & van Duijn, 1992; H. Liu, 2007; Magnus & Thissen, 2017; Rabe-Hesketh et al., 2004; Shiyko et al., 2012; Wang, 2010). Fully parametric MML via numerical integration can yield consistent and asymptotically unbiased parameter estimates, even when data are missing at random (MAR; Rubin, 1976) or otherwise unbalanced (e.g., Asparouhov & Muthén, 2012; De Boeck & Wilson, 2004; Gunes & Chen, 2014; H. Liu, 2007; Rabe-Hesketh et al., 2004; Vonesh, 2012) or when counts are small or underdispersed ( and therefore manifestly more discrete; Vonesh, 2012). In addition, MML permits the computation of likelihood-based information criteria (e.g., the LRT, AIC, and BIC), which remain the most powerful diagnostic tools available for use with multivariate count data, greatly facilitating the detection of model-data misfit (e.g., Magnus & Thissen, 2017; Vonesh, 2012). Lastly, although MML estimation via numerical integration can be computationally intensive, it need not be prohibitively so. Smith & Blozis (2014), for example, demonstrate that very few quadrature nodes may be needed, especially when adaptive Gauss–Hermite (AGH) quadrature is used.

## 4 Empirical application: Modeling morpheme counts

Speech-language pathologists often analyze the number of individual BGMs produced in an oral language sample to identify specific grammatical targets for clinical intervention (e.g., Bland-Stewart & Fitzgerald, 2001; Paul & Alforde, 1993; Tommerdahl & Kilpatrick, 2014). To this end, we demonstrate the feasibility and utility of fitting the proposed first-order multiphase SLCM to germane empirical data by applying the model to counts of Brown’s (1973) second grammatical morpheme (BGM2) “in” (e.g., “Juice **in** cup”; Table 1) collected in the longitudinal assessment of GAE morphosyntactic development in young children who are typically developing with respect to expressive language. We estimate the population average trajectory to describe expected development with respect to use of the morpheme “in”. We estimate individual trajectories to demonstrate how comparing individual curves to the population average curve can help inform inferences about individual development. By examining unexplained variability in use of the morpheme “in” over the course of early childhood among typically developing young children, we additionally demonstrate how the *unexplained variability* with which this morpheme is used appears to track inversely with chronological age and the *frequency* with which it is used, potentially signalling *acquisition*, which Brown (1973) defined as occurring when a child knows *when* and *how* to use a particular morpheme type.

### 4.1 Data

Expressive language data were taken from oral language sampled from 1,084 typically developing monolinguistic native speakers of GAE aged 1.5 to 5 years, inclusive (i.e., aged 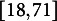 months), drawn from across 23 corpora in the Child Language Data Exchange System (CHILDES; MacWhinney, 2000), accessible at https://childes.talkbank.org/. Sample characteristics are provided in Table 3.

The number of children sampled from each corpus varied notably across corpora. The percentages of male and female children also varied across corpora but were comparable in the overall sample (46.86% male, 45.20% female, and 7.93% not reported). Oral language was sampled through engagement in either a toy play, narrative, group, book, or meal activity, where the range of activities varied across and sometimes also within corpora. The number of sampled utterances (exposure) varied across corpora, children within corpora, and assessments within child. Sampled assessments were required to contain a minimum of 25 utterances of sufficient quality (i.e., at least 25 “mean length of utterance (MLU)-eligible” utterances) to ensure a child had sufficient opportunity to demonstrate his/her level of morphosyntactic development. No notable relationship was discerned between a child’s chronological age in months (strongly related to level of morphosyntactic development in typically developing young children) and the number of sampled utterances in either the overall sample or within corpora (Figure 4), facilitating stable model estimation, the selection of an appropriate model (through stable parameter estimates, accurate standard errors, and the resulting apparent significance of individual parameter estimates), and the interpretation of estimated growth parameters as intended.

### 4.2 Analysis

Expressive language data were retrieved from North American English corpora in CHILDES (see Table 3) at TalkBank.org and extracted using Computerized Language ANalysis (CLAN) software (MacWhinney, 2000). To inform the selection of an appropriate conditional response distribution (and thus measurement model), several analyses were conducted, where candidate distributions included the Poisson and NB2.^2^ First, the empirical mean and variance of the number of “in” morphemes produced were computed within each 3-month age interval and plotted over time (i.e., over the course of early childhood) to check for empirical under-, equi-, or overdispersion. Second, Poisson and NB2 models were fit to the cross-sectional data within each 3-month age bracket in R (R Core Team, 2023) using the glm function in the *stats* package and glm.nb function in the *MASS* package (Venables & Ripley, 2002), respectively, with the number of sampled utterances (the exposure) included in the linear predictor of the mean response (Equation (14)). The relative fit of the Poisson and NB2 models was evaluated by visually comparing corresponding KLD, Pearson, AIC, and BIC statistics within each 3-month age interval. Note that likelihood-based information criteria are being used to compare the overall fit of the Poisson and NB2 models instead of the LRT as the Poisson distribution is not a special case (i.e., a constrained version) of the NB2 distribution. Rather, it is a limiting distribution as the strictly positive NB2 dispersion parameter tends to zero (e.g., Casella & Berger, 2002). 
(14)

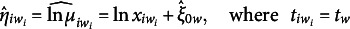



After selecting a measurement model, scatterplots and cubic smoothing splines of the cross-sectionally estimated model-implied mean log expected BGM2 counts and, if applicable, dispersion parameters were plotted over time to inform the selection of the functional form of the population average trajectory and, if applicable, the dispersion trajectory, respectively. A multiphase SLCM was subsequently fit to the longitudinal data in M*plus* ^©^ Version 8.5^3^ using MML estimation with parameter estimates and corresponding standard errors robust to both nonnormality of the count responses and dependence among observations by specifying TYPE=COMPLEX and ESTIMATOR=MLR in the ANALYSIS command in conjunction with use of the CLUSTER option in the VARIABLE command (Muthén & Muthén, 2017). Robust standard errors were computed using a sandwich estimator and—by default—the observed information matrix evaluated at the maximum likelihood estimates of model parameters (i.e., the Hessian matrix; Muthén & Muthén, 2017). MML estimation was implemented via the Expectation-Maximization (EM) algorithm (e.g., Bock & Aitkin, 1981) with adaptive Gauss-Hermite quadrature (e.g., Cai, 2010; Rabe-Hesketh et al., 2004; Schilling & Bock, 2005; Vonesh, 2012; Wang, 2010) with the default of 15 integration points per dimension of integration.

Because the number of sampled utterances (the exposure) varied across children and assessments within child, each measurement model fit to BGM2 counts throughout this analysis included an offset in the linear predictor of the mean response (the expected BGM2 count) constructed as the natural log of the number of utterances sampled from a child at an assessment with regression coefficient fixed at one. An alpha level of 0.05 was used for all hypothesis tests. All tables and figures were produced in R version 4.3.1 (R Core Team, 2023). Likewise, all data processing/management and descriptive and exploratory analyses were conducted in version 4.3.1 (R Core Team, 2023). M*plus* ^©^ model results were harvested and read into R using the *MplusAutomation* package (Hallquist & Wiley, 2018) and analyzed using the *MASS* package (Venables & Ripley, 2002). All datasets, computer code, and Supplementary Material are provided on the OSF website for this project at: https://osf.io/j6rp7/?view_only=c6a9f74add0d476dbeb1e7abd6a76cb2.

### 4.3 Results

Results are reported in the order in which they were obtained, as we build the complete model from the ground up—that is, from the observed data (e.g., chronological ages, BGM2 counts, sampled utterance counts) up to the hypothesized data-generating latent structure, where the observed and latent variables are connected by measurement models—and then proceed to make inferences based on the final, full model. First, we select appropriate measurement and structural models, where the latter includes trajectories for both the mean (expected count) and dispersion. Second, we demonstrate identification of the complete model. Third, we discuss individual and population-level inferences about expressive language development based on the fitted model.

#### 4.3.1 Measurement model

Recall that measurement invariance is assumed across individuals and occasions. One key aspect of ensuring measurement invariance over the chronological ages of assessment is specifying the same measurement model in each 3-month age interval. As such, when selecting an appropriate measurement model, we must consider both theoretical (e.g., clinical) justification and empirical fit across the entire span of early childhood (i.e., between 1.5 and 5 years of age, inclusive).

**Figure 5:**
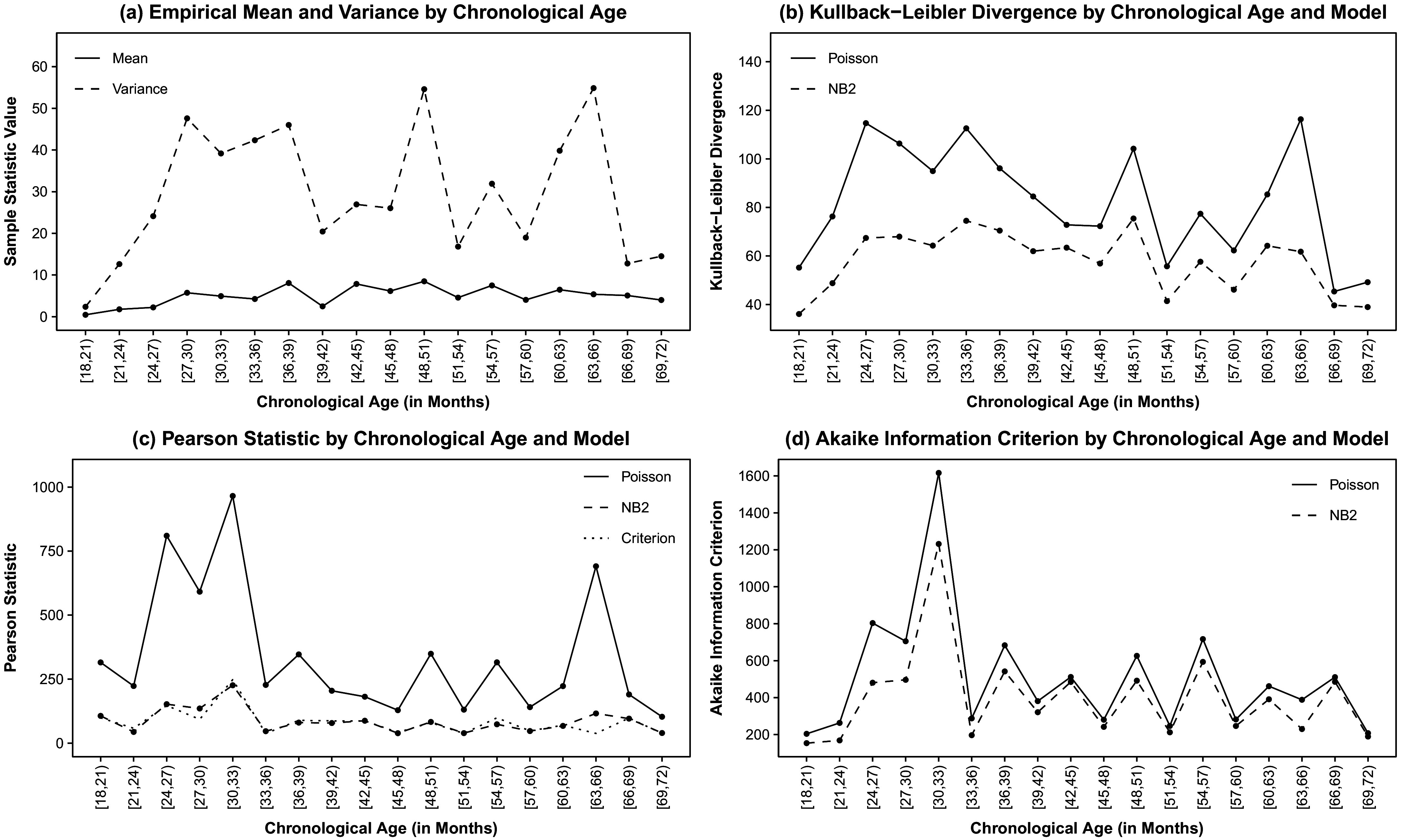
Model-data fit evaluations conducted to inform measurement model selection.*Note*: Sample characteristics are provided in Table 3.

First, visual inspection of the empirical mean and variance over time revealed empirical overdispersion within each 3-month age interval (Figure 5a). Second, the KLD for the NB2 model was notably lower than that of the Poisson model, suggesting the NB2 model consistently better described the shape of the conditional response distribution (i.e., provided a better fit to the data; Figure 5b). Third, the Pearson statistic for the Poisson model was notably higher in each 3-month age interval than the corresponding degrees of freedom (see, e.g., Cameron & Trivedi, 2013), suggesting the presence of substantial overdispersion under the Poisson model (Figure 5c). In contrast, the Pearson statistic for the NB2 model and the corresponding degrees of freedom were closely aligned over the course of early childhood, suggesting the NB2 model capably captured both the mean and variability in observed BGM2 counts across the chronological ages of assessment (Figure 5c). Note that the degrees of freedom for the Poisson and NB2 models in a given 3-month age interval differ only by one (for the NB2 dispersion parameter)—a difference that cannot be readily discerned in Figure 5c given the *y*-axis scale. As such, to make Figure 5c easier to visually decipher, only the degrees of freedom (i.e., the “criterion”) for the NB2 model are plotted as the degrees of freedom for the Poisson model are practically overlapping for a given age bracket.

Fourth and lastly, Akaike and Bayesian information criteria were compared between the Poisson and NB2 models in each 3-month age interval. Figure 5d shows the AIC values obtained under the Poisson and NB2 models in each 3-month age interval. Since almost identical results were obtained using the BIC as only a single additional parameter is estimated under the NB2 model, this plot has been omitted. As can be seen in Figure 5d, the NB2 model yields a lower AIC value (better overall fit) in each 3-month age interval except for the last one, in which the Poisson and NB2 models appear to provide comparable overall fit to the data. Note that the differences between these two models do not seem quite as drastic when using these measures of relative overall fit as compared to the more targeted investigations reported earlier evaluating the ways in which the Poisson and NB2 distributions meaningfully differ (i.e., in terms of the model-implied variance and shape).

Collectively, these findings suggested the NB2 model provided a better fit to the BGM2 counts in each 3-month age interval than the Poisson model. The NB2 model also had the additional appeal of permitting the investigation of concurrent changes in the *frequency* (quantified by the mean) and *unexplained variability* (quantified by the dispersion parameter) with which typically developing children produce the morpheme “in” over the course of early childhood, where collective change in *explained use* may reflect *correct use* and *acquisition*. As such, BGM2 counts were assumed to conditionally follow a NB2 distribution within each 3-month interval (Equation (15)). 
(15)

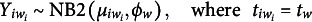



#### 4.3.2 Mean trajectory

Visual inspection of individual empirical trajectories of the rate at which the morpheme “in” is produced within an oral language sample and the cubic smoothing spline fit to the overall sample suggested *frequency of use* may follow a linear–linear trajectory over the course of early childhood with the transition from phase 1 to phase 2 occurring somewhere between 27 and 36 months of age, confirming Brown’s (1973) observations based on only three children (Figure 2). Similarly, subsequent visual inspection of the scatterplots and cubic smoothing splines of the cross-sectionally estimated NB2 mean log expected BGM2 production rate over time ( 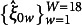 in Equation (14)) also suggested a linear–linear trajectory over the course of early childhood, with a continuous, smooth/gradual transition from phase to phase at the population level occurring somewhere between 27 and 39 months of age (Figure 1). This apparent linear–linear development process aligns with the linear–linear process by which GAE grammar is understood to develop in typically developing young children, with an initial phase of rapid development (phase 1) followed by a period of slower, sustained development (phase 2), with a continuous, smooth/gradual transition from phase-to-phase and where the age of transition from phase 1 to 2 may vary widely. Moreover, the apparent transition age range of 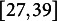 months encompasses the typical age of acquisition of 27 to 30 months posited by Brown (1973) (see Table 1).

Note that a quadratic trajectory was not considered in this case due to challenges with interpreting quadratic functions, including the unrealistic implication that typically developing children decline in level of morphosyntactic development (and thus also the rate at which the morpheme “in” is produced within an oral language sample) after a certain chronological age. Other potentially salient functions for the mean trajectory include negative exponential (e.g., Sterba, 2014), Jenss–Bayley (e.g., J. Liu, 2022), and Gompertz functions. However, one of the limitations of these alternative functions is the lack of freely estimated changepoints. As previously discussed, quantifying the population average age of transition from an initial phase of rapid development to a subsequent period of more gradual, sustained development and mastery in typically developing children may help inform when children ought to be assessed for expressive language disorders, such as late language emergence; and comparing a child’s individual trajectory and changepoint to the population average trajectory and changepoint among typically developing children may help identify children who fall below age expectations and warrant further clinical evaluation, intervention, and monitoring.

A linear–linear LGM was therefore considered for the log expected counts in which all growth parameters were allowed to vary across individuals due to the high degree of variability in expressive language development clinically expected among typically developing children. The model for individual *i* to be fit to the data is comprised of linear predictor 
(16)

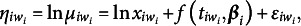

where 

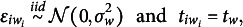

with linear–linear growth function 
(17)

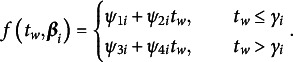



It is presumed, at least initially, that an individual’s growth parameters are the sum of fixed and random effects 
(18)

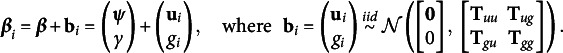



The linear–linear trajectory in Equation (17) contains a total of five freely estimated individual growth parameters: four that enter the function linearly, 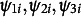 , and 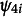 , and one, the changepoint 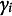 , that enters the function in a nonlinear fashion.

Exponentiated phase 1 intercept 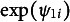 captures the rate at which child *i* is expected to produce the morpheme “in” within an oral language sample at age 18 months. Shifted changepoint 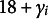 quantifies the chronological age (in months) at which child *i* is expected to transition developmentally from phase 1 (rapid development) to phase 2 (slower, sustained development). Exponentiated phase 2 intercept 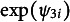 captures the rate at which child *i* is expected to produce the morpheme “in” within an oral language sample at anticipated transition age 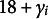 months. Lastly, the rate at which child *i* is expected to produce the morpheme “in” within an oral language sample increases multiplicatively by an order of 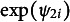 with every 1 month increase in chronological age between 18 and 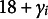 months, inclusive, and by an order of 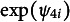 with every 1 month increase in chronological age after age 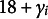 months.

The population growth parameters 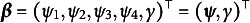 are interpreted similarly to the corresponding individual growth parameters and describe the population average trajectory for typically developing monolinguistic native speakers of GAE aged 1.5 to 5 years, inclusive. For example, exponentiated phase 1 intercept 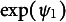 captures the rate at which typically developing children are expected to produce the morpheme “in” within an oral language sample at age 18 months, on average, while shifted changepoint 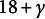 quantifies the chronological age (in months) at which typically developing children are expected to transition developmentally from phase 1 to phase 2 on average. Random effects 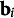 are assumed to jointly follow a multivariate normal distribution with zero mean vector and unstructured, symmetric covariance matrix, 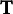 (Equation (18)).

A continuous though abrupt transition from phase 1 to phase 2 was created by imposing zero-order continuity (Cudeck & Klebe, 2002) at the changepoint, although examination of Figures 1 and 2 suggested a smooth/gradual transition from phase to phase. Zero-order continuity is the highest order of continuity that can be imposed at the knot when a first-order polynomial is used to describe growth in each adjacent phase. This restriction permits the elimination of one linear growth parameter from the set of freely estimated model parameters for child *i*. Phase 2 intercept 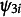 was selected for elimination in this case as its interpretation is less clinically useful than that of the other linear growth parameters. 

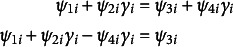



The resulting trajectory is provided in Equation (19). Note that 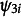 can readily be computed using estimates of the other individual growth parameters. 
(19)

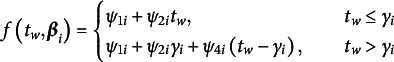



The linear–linear trajectory for child *i* now contains only four freely estimated individual growth parameters: 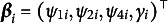 . The random effects covariance matrix 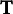 is likewise reduced to 
(20)

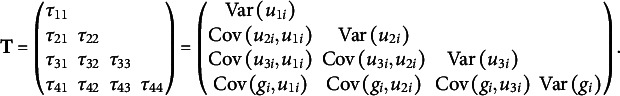



The nonlinear growth model in Equation (19) can be reformulated as an SLCM by taking by taking a first-order Taylor series expansion around the population growth parameters and setting the population average changepoint to zero ( 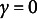 ): 
(21)

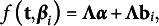

where the 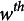 row of factor loading matrix, 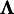 , is 
(22)

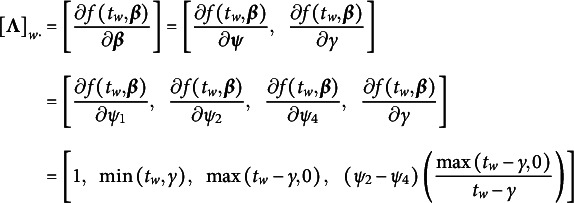



Here, the minimum and maximum functions (Seber & Wild, 2003) 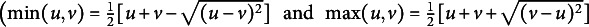 , where *u* and *v* are real numbers) are utilized and directly coded into the factor loading matrix.

#### 4.3.3 Dispersion trajectory

Having specified a structural model (in this case, an SLCM) describing change in the log expected rate of “in” morpheme production over the course of early childhood, we now turn our attention to the time-varying NB2 dispersion parameters. Recall that the NB2 dispersion parameter in a given 3-month age interval quantifies the level of variability in BGM2 counts *unexplained* by the mean (and predictors thereof) *across* the individuals assessed within that age window. The dispersion parameters are not individual-level parameters but population-level parameters that may vary over time and perhaps even follow a recognizable functional form across chronological age. As such, the trajectory specified for the dispersion parameters does not have parameters that vary across or within individuals (and thus no random effects and variance components). Instead, there is only the population-level trajectory to be specified by imposing constraints on the time-varying dispersion parameters.

Recall also that visual inspection of the scatterplots and cubic smoothing splines of the cross-sectionally estimated NB2 dispersion parameter ( 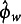 ) over time suggested the dispersion parameters follow a linear–linear trajectory with a continuous, smooth transition from phase to phase at the population level that mirrors (from below) the population average trajectory in expected BGM2 counts over the course of early childhood (Figure 1). Initially, a linear function was fit to the natural log of the NB2 dispersion parameters (Equation (12)). This function is highly parsimonious while also capturing many key features of the change in dispersion over time—the steep decline prior to 40 months of age, the leveling off thereafter, and the smooth transition from the former phase to the latter. However, fitting the regression model in Equation (12) to the time-varying dispersion parameters yielded a decline prior to age 40 months that was too gradual and, critically, an asymptote of zero, which is not compatible with the parameter space of the strictly positive NB2 dispersion parameter. Thus, alternatives were considered. An exponential decay function with a nonzero asymptote (Equation (13)) was ultimately selected due to its parsimony (only 3 parameters), interpretability (as previously discussed), and capability of capturing *all* salient features of the change in dispersion over time: a precipitous decline prior to 40 months of age, the leveling off thereafter to a nonzero asymptote, and the smooth transition from the former phase to the latter.

#### 4.3.4 Model identification

The mean structure of the resulting first-order linear–linear SLCM consists of the four freely estimated growth factor means ( 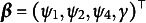 in Equation (18)), while the covariance structure contains a total of 13 free parameters—10 growth factor variances and covariances (unique elements of 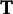 in Equation (20)) and 3 parameters for the exponential decay trajectory with nonzero asymptote fit to the time-varying NB2 dispersion parameters ( 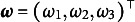 in Equation (13)). With 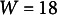 unique measurement occasions in the overall sample of 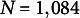 children, the mean structure is overidentified ( 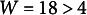 ) and the covariance structure is overidentified ( 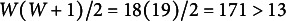 ). As such, the model as a whole is overidentified, permitting us to obtain a unique set of parameter estimates and to meaningfully evaluate model fit (e.g., Kline, 2016).

The final overall model was fit to the data with the trajectory for the NB2 dispersion parameters in Equation (13) fit by imposing model constraints on the freely estimated time-varying dispersion parameters. Note that using model constraints to fit the exponential decay trajectory to the time-varying dispersion parameters changes their estimated values while also notably reducing the number of freely estimated parameters in the covariance structure. Note also that although the fitted first-order linear–linear NB2 SLCM is overidentified in the overall sample, for this and other LGMs, empirical identification may be compromised depending on the number of assessments and ages of assessment per child. As in most real-world observational studies conducted using young children, there is a fair amount of missing data in the overall sample. In this case, much of this “missingness” is an artifact of (a) the misalignment among corpora in planned ages of assessment and (b) children within a corpus sometimes being assessed at slightly different ages than were planned. 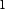 Population parameter estimates, standard errors, and *p*-values are provided in Table 4.

**Table 4:** Population parameter estimates for NB2 first-order linear-linear SLCM fit to longitudinal BGM2 counts

Parameter	Estimate	Standard error	*p*-value
Population average phase 1 intercept ( 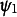 )	-5.799	0.595	<0.001
Population average phase 1 slope ( 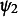 )	0.246	0.057	<0.001
Population average phase 2 intercept ( 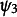 )	-3.402	0.246	<0.001
Population average phase 2 slope ( 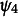 )	0.006	0.008	0.469
Population average changepoint ( 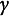 )	9.958	0.922	<0.001
Expected BGM2 production rate at age 18 months ( 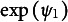 )	0.003	0.002	0.093
Multiplicative increase in BGM2 production rate per month in phase 1 ( 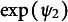 )	1.279	0.073	<0.001
Expected BGM2 production rate at population average transition age ( 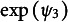 )	0.033	0.008	<0.001
Multiplicative increase in BGM2 production rate per month in phase 2 ( 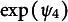 )	1.006	0.008	<0.001
Variability in phase 1 intercept ( 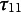 )	0.983	0.786	0.211
Variability in phase 1 slope ( 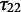 )	0.004	0.006	0.470
Variability in phase 2 slope ( 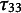 )	0.000	0.000	0.441
Variability in changepoint ( 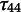 )	0.558	1.614	0.730
Covariance between phase 1 intercept and phase 1 slope ( 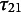 )	-0.060	0.071	0.402
Covariance between phase 1 intercept and phase 2 slope ( 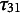 )	0.002	0.004	0.606
Covariance between phase 1 intercept and changepoint ( 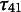 )	-0.465	0.653	0.477
Covariance between phase 1 slope and phase 2 slope ( 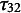 )	0.000	0.000	0.971
Covariance between phase 1 slope and changepoint ( 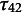 )	0.012	0.044	0.776
Covariance between phase 2 slope and changepoint ( 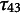 )	-0.003	0.007	0.644
Dispersion trajectory unadjusted intercept ( 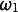 )	3.541	0.000	<0.001
Dispersion trajectory decay factor ( 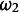 )	0.837	0.029	<0.001
Dispersion trajectory nonzero asymptote ( 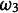 )	0.205	0.067	0.002

*Note*: BGM2 denotes Brown’s (1973) second grammatical morpheme, “in” (see Table 1). Sample characteristics are provided in Table 3.

**Figure 6:**
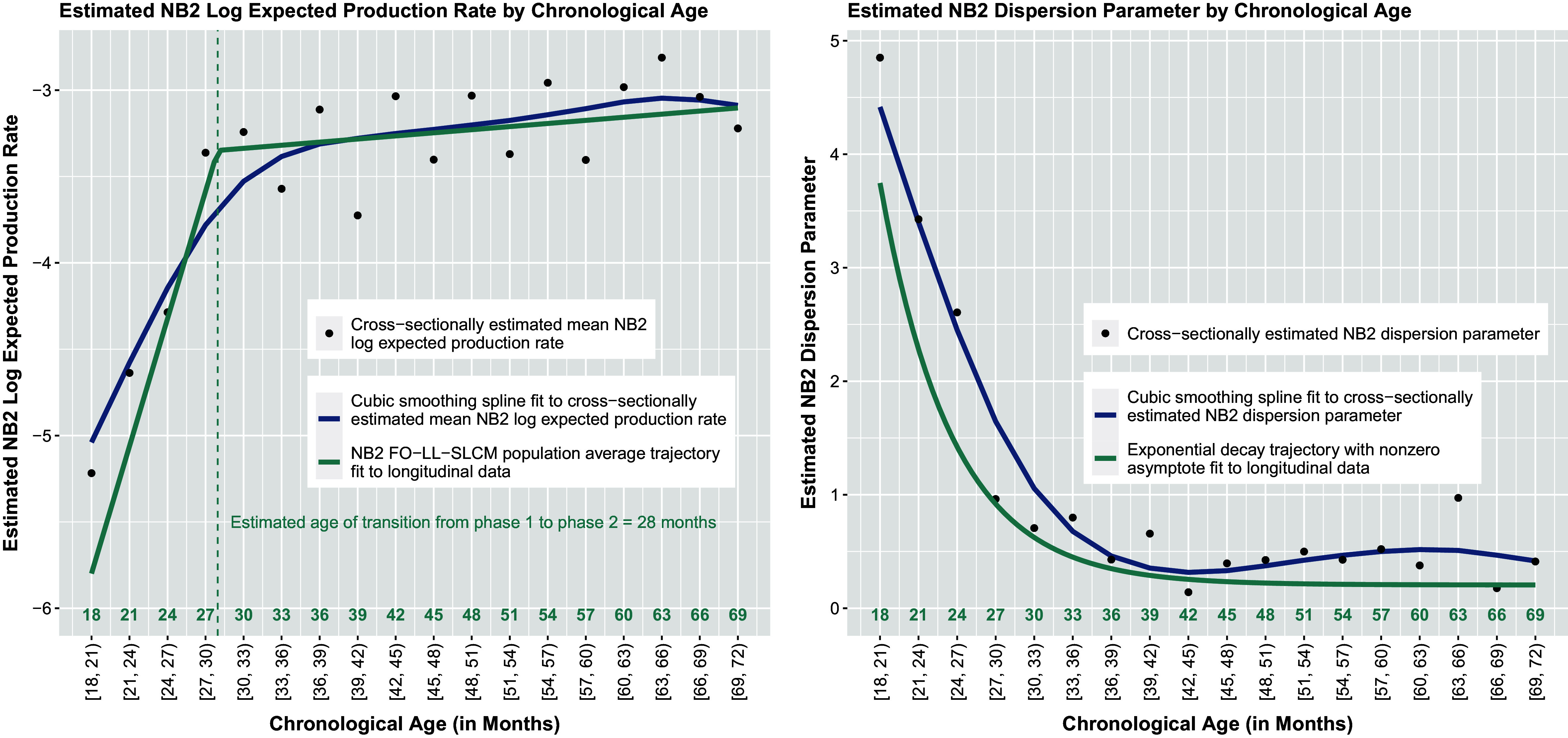
NB2 log expected BGM2 production rate and dispersion by chronological age. *Note*: BGM2 denotes Brown’s (1973) second grammatical morpheme, "in” (see Table 1). NB2 denotes the Negative Binomial distribution with mean 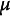 , dispersion 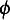 , and quadratic variance function 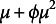 . FO-LL-SLCM denotes the first-order linear–linear structured latent curve model fit to the data, yielding the population parameter estimates in Table 4. Sample characteristics are provided in Table 3.

The population average trajectory for the log expected rate of “in” morpheme production and the exponential decay trajectory fit to the dispersion parameters are provided in Figure 6.

#### 4.3.5 Model-implied development at the population level

Looking at the left-most panel of Figure 6, one can see the *frequency* with which typically developing monolinguistic native speakers of GAE produce the morpheme “in” increases rapidly between 1.5 and 2.5 years of age but then levels off. More specifically, on average, typically developing children are expected to produce approximately 3 “in” morphemes within an oral language sample of 1,000 utterances at age 18 months (since 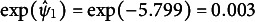 ), transition from rapid development (phase 1) to slower, sustained development (phase 2) at age 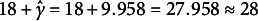 months, and produce about 33 “in” morphemes within an oral language sample of 1,000 utterances at the expected age of transition from phase 1 to phase 2 of 28 months (since 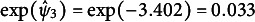 ) (see Table 4). On average, for every additional one month increase in chronological age between ages 18 and 28 months, inclusive (i.e., during developmental phase 1), the rate at which “in” is produced in an oral language sample is expected to increase multiplicatively by an order of 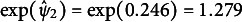 . In contrast, the rate at which “in” is produced is not expected to change with increasing chronological after age 28 months (i.e., in developmental phase 2, since 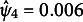 with 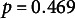 ).

With that said, the log expected rate at which the morpheme “in” is produced within an oral language sample does not significantly vary across children at age 18 months ( 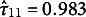 , 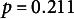 ). Likewise, the chronological age at which children transition from phase 1 to phase 2 does not significantly vary ( 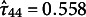 , 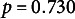 ), and the rate of change in the log frequency with which the morpheme “in” is produced does not significantly vary across children in either phase 1 ( 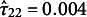 , 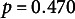 ) or phase 2 ( 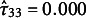 , 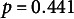 ). Moreover, none of the four freely estimated growth factors (phase 1 intercept and slope, phase 2 slope, and changepoint) significantly covary (i.e., all off-diagonal elements of 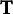 are essentially zero).

Simultaneously, looking at the right-most panel of Figure 6, *unexplained variability* in use of the morpheme “in” drops dramatically between 1.5 and 2.5 years of age and then remains low. More specifically, the estimated coefficients of the exponential decay function fit to the time-varying NB2 dispersion parameters imply the dispersion parameter is expected to be 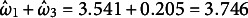 at age 18 months ( 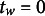 ), decrease by 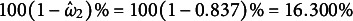 with every 1 month increase in chronological age, and approach nonzero asymptote 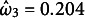 as children reach the end of early childhood at 5 to 6 years of age.

Collectively, these population level trajectories suggest acquisition of the morpheme “in” might be evidenced, among typically developing monolinguistic native speakers of GAE aged 1.5 to 5 years, inclusive, by an increase in *explained use*, which may prove to be a facile manifestation of *correct* use. Moreover, the statistical insignificance of the growth factor variances and covariances suggests the process by which children learn to use the morpheme “in” may be highly consistent among typically developing young children. This consistency among *typically* developing children may facilitate the identification of *atypically* developing children when comparing individual fitted curves to the population average curve quantifying age expectations in typical expressive language development.

#### 4.3.6 Model-implied individual development

**Figure 7:**
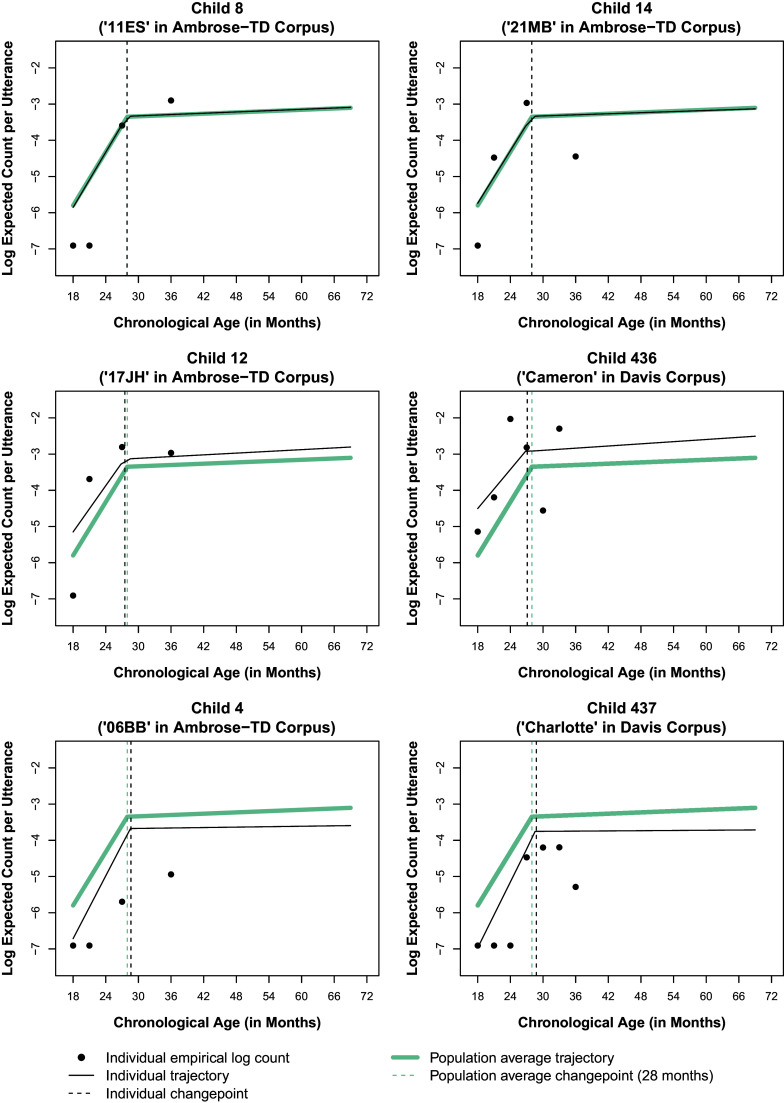
Example individual fitted trajectories.

Example individual fitted trajectories are provided in Figure 7. Although generally the functional form of the fitted individual trajectories need not be the same as that of the population average trajectory (S. A. Blozis & Harring, 2015), in this particular case both are linear–linear (Figures 6 and 7). An individual’s fitted trajectory describes that individual’s model-implied production of the morpheme “in” over the course of early childhood. These individual fitted trajectories can be compared to the population average trajectory to help support clinical inferences about individual development and to potentially help identify children who may benefit from interventions targeting use of the morpheme “in” in GAE. For example, failure to observe any use of the morpheme “in” in an oral language sample of 100 utterances after age 28 months should motivate a closer examination of the child for high suspicion of language delay.

For example, the model-implied trajectories for children 8 and 14 in Figure 7 imply these children are developmentally on track (i.e., developing according to age expectations throughout early childhood). Alternatively, the model-implied trajectories for children 12 and 436 in Figure 7 imply these children are developing slightly above age expectations throughout early childhood—although not to a degree that is clinically meaningful as all sampled children were considered to be typically developing with respect to expressive language at the time(s) of assessment. This above average production of the morpheme “in” appears to be largely driven by these two children *entering* early childhood with a higher-than-average production rate despite subsequent slower than average increases in production during the initial phase of rapid development (phase 1). Indeed, of the 453 (41.79%) children in the overall sample whose fitted trajectories imply they are producing “in” more frequently than average when they enter early childhood, the vast majority (93.38%) subsequently experience slower than average growth in phase 1 (though generally not slow enough to fall to or below age expectations). In contrast, the model-implied trajectories for children 4 and 437 in Figure 7 imply these children are developing slightly below age expectations throughout early childhood (although, again, not to a degree that is clinically meaningful as all sampled children were typically developing). This below average production of the morpheme “in” appears to be largely driven by these two children entering early childhood producing fewer “in” morphemes than average despite subsequent faster than average increases in production during phase 1. Indeed, of the 630 (58.12%) children in the overall sample whose fitted trajectories imply they are producing fewer “in” morphemes than average as they enter early childhood, the vast majority (99.21%) experience faster than average growth in phase 1, though generally not fast enough to catch up to age expectations.

Collectively, these findings would seem to suggest that for typically developing children, the frequency with which a child produces “in” within an oral language sample at age 18 months may predict the frequency with which this morpheme is used for the duration of early childhood (and possibly beyond). Combined with the potential for issues with the production of “in” to foreshadow broader issues with a child’s overall level of expressive language development as well as issues using more strictly grammatical (as opposed to lexical) morphemes that are typically acquired later in childhood (e.g., Clark, 1973; Morgenstern & Sekali, 2009), use of the morpheme “in” in early childhood may be a fairly efficient, accessible, and early *proxy* for the child’s overall level of expressive language development. (In clinical science, proxies are quite useful as they are easier to measure but predict clinical outcomes of interest.) This may permit clinicians to more effectively target early interventions for children at risk for language delay—a significant early warning symptom of a wide variety of developmental disorders (e.g., Roberts et al., 2023).

## 5 Discussion

In the social and behavioral sciences, latent growth modeling, or one of its many variants, for continuous repeated measures data remains a predominant modern approach to better understand developmental processes thought to undergird many human behaviors, traits and attributes. Research studies employing LGMs often share common research objectives. Particularly, to gain an understanding of typical behavior of the underlying phenomena as represented by the parameters of a model, to assess the degree to which these parameters and hence the phenomena vary across individuals, and to investigate the extent that this variation can be explained by individual characteristics. Despite its popularity coupled with an abundance of available categorical data, applications of latent growth modeling utilizing discrete data are disappointingly rare.

The primary purposes of this article were to introduce a first-order multiphase SLCM for count response data and to apply the model to grammatical morpheme counts, a clinical measure of expressive language development. The growth parameters of the model—including changepoints—were unknown and allowed to vary across individuals, and exposure was permitted to vary across both individuals and time/assessments. Although typical acquisition of the morpheme “in” appeared to follow a linear–linear trajectory in the empirical example, the proposed model provides much more modeling flexibility, permitting non-monotonic change over the entire measurement period that may occur in more than two phases, where change within a given phase may be non-polynomial (Harring et al., 2021). We also demonstrated how to incorporate a trajectory describing concurrent change in time-varying dispersion (unexplained variability in morpheme counts) over the course of early childhood to provide additional insights into acquisition. We presented the motivating clinical context for the proposed model, the empirical data, analytic challenges and considerations, and analysis results. We demonstrated how to estimate the proposed model using existing methods and software, highlighting particular decision points as we stepped through the analysis.

Other methodological articles have also recognized the challenges with analyzing multivariate count data and have sought to help bridge the gaps between theoretical model development and their applications to real-world data. For example, in a recent article, Seddig (2024) showed how to fit first-order LGMs to longitudinal count response data and over-dispersed, zero-inflated response data using M*plus* ^©^. Seddig (2024) focused attention on model specification using the software, parameter interpretation, and model selection using global fit statistics and model comparison procedures.

We extended the basic models presented by Seddig (2024) in numerous ways. For example, Seddig (2024) limited discussion to polynomial growth functions with examples restricted to linear and quadratic trajectories with growth parameters interpreted with respect to post-baseline measurement occasion rather than time (e.g., age) itself. Varying exposure was not discussed nor was the possibility of specifying a trajectory for time-varying/time-specific dispersion parameters. We note that the growth trajectory presented for the inflation factor in zero-inflated count models is not analogous to a dispersion parameter trajectory because the dispersion parameter impacts the variance but not the mean of the count distribution and is defined across individuals at a given measurement occasion so that the trajectory is defined only at the population level (as constraints on the time-varying dispersion parameters).

We extend the growth models to include nonlinear functions such as the bilinear model. The linear–linear function discussed in this article need not be monotonic nor defined by a polynomial within a given phase, with freely estimated linear and nonlinear growth parameters (including changepoints) that may vary across individuals, an exposure variable in the linear predictor of the mean response, and factor loadings constructed as functions of time and freely estimated growth parameters (as per Harring et al., 2021), permitting unequally spaced and potentially individual measurement occasions and yielding growth parameters that are interpreted with respect to time itself rather than measurement occasion. As previously noted, few examples exist in the literature of a nonlinear growth function of linear and nonlinear random effects connected to observed counts via a nonlinear link function due to the computational difficulties that may arise in model estimation. In this article, we offer one such example by fitting the proposed first-order SLCM to longitudinal morpheme counts.

Of course, even the first-order LGM model we presented can be embellished and advanced in several ways including (1) incorporating both observed and latent time-invariant covariates to explain differences in characteristics of growth (i.e., the parameters defining the linear–linear growth model), (2) adding individual attributes to account for systematic associations inherent in the modeling of growth and/or the dispersion parameter, and (3) relating growth characteristics to some distal outcome measure that might be predictive of grammatical morpheme development. One interesting extension would permit a nonlinear mixed effects model (Davidian & Giltinan, 2003; Vonesh, 2012) to be fitted as the growth modeling framework. This type of GLMM could be fitted within the functionality of SAS NLMIXED or in modules in R (see, e.g., Grimm & Stegmann, 2019, for a fairly comprehensive listing).

**Figure 8:**
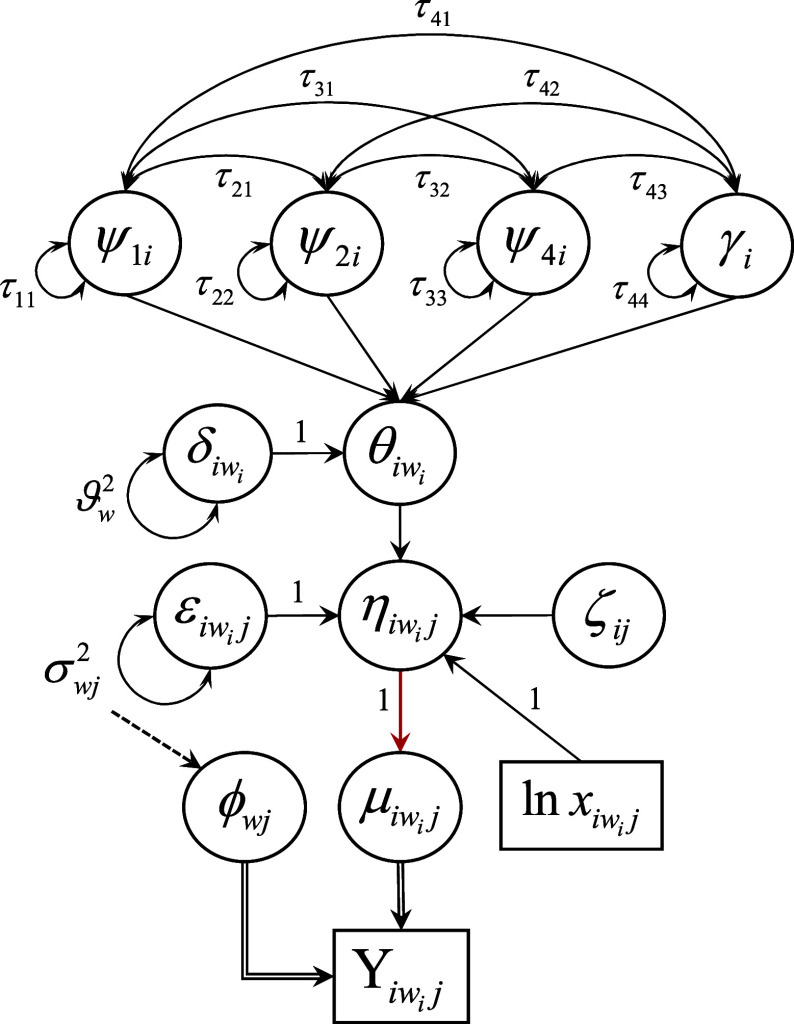
Path diagram for three-tier formation of a second-order linear–linear latent growth model fit to repeated measurements of multiple count response variables that each conditionally follow a 2PEF distribution at each measurement occasion. *Note*: Much of the notation used in Figure 8 is the same as the notation used in Figure 4. A solid, single-line, red arrow indicates a structural relationship with a non-identity (e.g., natural log) link function. A dashed black arrow from *A* to *B* indicates *A* gives rise to *B* directly and/or indirectly. A solid, double-line, black arrow from *A* to *B* indicates *A* generates *B*.

**Figure 9:**
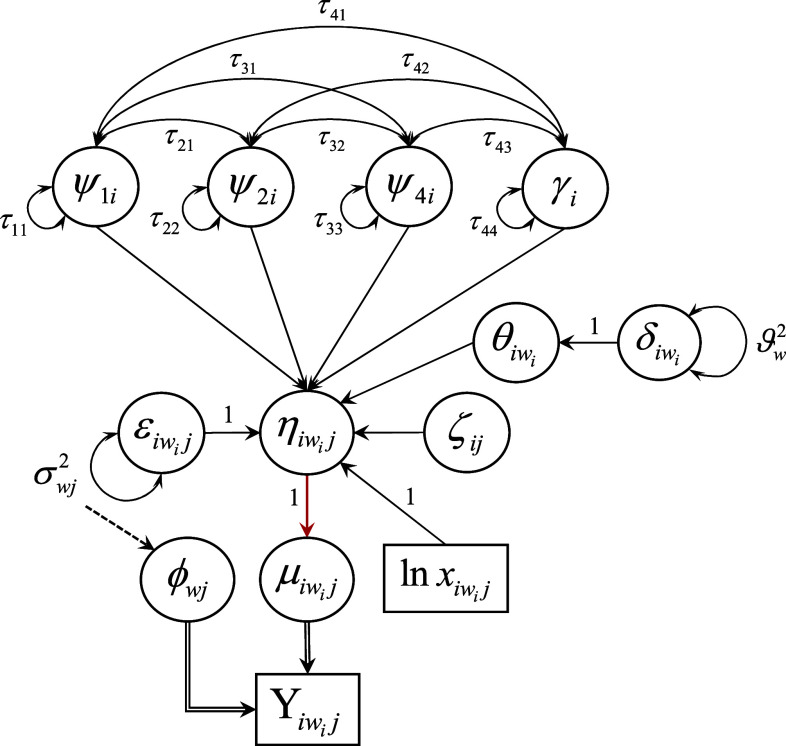
Path diagram for two-tier formation of a second-order linear–linear latent growth model fit to repeated measurements of multiple count response variables that each conditionally follow a 2PEF distribution at each measurement occasion. *Note*: Much of the notation used in Figure 9 is the same as the notation used in Figure 4. A solid, single-line, red arrow indicates a structural relationship with a non-identity (e.g., natural log) link function. A dashed black arrow from *A* to *B* indicates *A* gives rise to *B* directly and/or indirectly. A solid, double-line, black arrow from *A* to *B* indicates *A* generates *B*.

Multiple measures (or items) are sometimes present at each measurement occasion because they are believed to be indicators of the same latent construct (i.e., counts of multiple morphemes thought to measure expressive language). In this scenario the researcher is likely to be interested in change in the latent construct underlying those measures rather than the measures themselves. In such cases, one may analyze second-order LGMs (Hancock et al., 2001; Sayer & Cumsille, 2001). For example, Figure 8 shows a path diagram of a second-order linear–linear LGM, which might be more feasibly estimated in two-tier formation using a Schmid–Leiman transformation (Figure 9; e.g., Cai, 2010; Rijmen, 2010; Schmid & Leiman, 1957).

The central differences between the second-order models depicted in Figures 8 and 9 and the first-order model in Figure 4 is that now inferences can be made about a child’s *level of GAE morphosyntactic development* overall rather than use of a specific morpheme. While the latter may be useful for identifying specific grammatical targets for clinical intervention, the former may be more helpful for identifying children who are developing atypically with respect to expressive language to help expedite treatment delivery for improved long-term outcomes. For example, to this end, Yang et al. (2022) fit linear–linear trajectories to score data from various measures of lexical diversity from typically developing (TD) and developmentally language delayed (DLD) children aged 2–6 years. Lastly, fitting a second-order model allows us to parse regression error in the latent construct arising from misspecification of the population growth curve or other aspects of the structural model ( 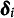 in Equation (4)) from error in the linear predictor ( 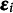 in Equation (3)) arising from the omission of and/or measurement error in predictors of the mean response. As such, *variability* in these two different levels/sources of error can be separated and freely estimated, which may be useful for substantive reasons but also permits interpretation of the dispersion parameters as quantifying specifically the latter source of error rather than any/all sources of regression error.

Over realistic spans of time, many human behaviors, traits, and attributes develop or change nonlinearly. At the heart of the SLCM is a nonlinear growth function whose parameters can be tied to, and often derived to characterize, interesting features of the development under investigation. We demonstrated how a theoretically driven linear–linear piecewise growth model could effectively summarize GAE morphosyntactic development captured by multivariate count data. An in-depth analysis was conducted with an emphasis on parameter interpretation, model-assessment, and refinement. Our primary purpose was not to make substantive claims regarding the findings on how the grammatical morpheme “in” changed over time, but rather, to suggest a road map to help researchers successfully navigate a fairly involved set of analytic activities with several junctures requiring thoughtful decision-making. Future directions include employing the proposed first-order SLCM to examine additional BGMs that are more closely associated with expressive language delay and chronic language disorder in early childhood, such as those marking tense (e.g., past tense markers), agreement (e.g., third person singular marker), and aspect (e.g., Leonard et al., 2004; Rice, 2003). Additionally, the second-order extension of the proposed model (discussed earlier) could be fit to counts of all 14 BGMs in the assessment of a child’s overall level of expressive language development to yield additional insights beyond what Brown (1973) was able to discover with only three children, including further quantitative evaluation of the extent to which a child’s use of “in” may be a proxy for overall level of expressive language development in early childhood.

## Data Availability

The data that support the findings of this study are openly available in the Child Language Data Exchange System (CHILDES) accessible at https://childes.talkbank.org/. CHILDES is supported by grant NICHD HD082736.

## Footnotes

1 Refer to the Supplementary Material available on the companion OSF website at: https://osf.io/j6rp7/?view_only=c6a9f74add0d476dbeb1e7abd6a76cb2.

2 Inflated and hurdle count models (such as Zero-Inflated Poisson and Negative Binomial Hurdle models) were not considered in this analysis as they suppose that at a certain level of morphosyntactic development a child will produce at least one “in” morpheme *regardless of how many utterances are sampled*. This assumption is not particularly tenable when one considers the impact of oral language sampling context on how many of which morphemes are produced and that the total number of morphemes a child can produce within a fixed number of utterances must be shared across morpheme types. In contrast, in this study, zero counts are considered to arise when a child’s level of morphosyntactic development lies below some latent threshold and/or an insufficient number of utterances has been sampled, where both may depend on the language sampling context. Dispersion parameters, on the other hand, were of clinical (substantive) interest as they permit one to investigate concurrent changes in the *frequency* and *unexplained variability* with which typically developing children produce the morpheme “in” over the course of early childhood, where collective change in *explained use* may reflect *correct use* and *acquisition*.

3 The proposed first-order multiphase SLCM for count response data involves a nonlinear growth function comprised of linear and nonlinear random effects connected to observed counts via a nonlinear link function, with an exposure that varies across both individuals and measurement occasions in the linear predictor of the mean response, and factor loadings constructed as nonlinear functions of population growth parameters and chronological age at time of assessment. M*plus* ^©^ Version 8.5 was used to fit this model to the empirical data due to its ability to handle all of these model features simultaneously.
